# Novel 2-substituted-benzimidazole-6-sulfonamides as carbonic anhydrase inhibitors: synthesis, biological evaluation against isoforms I, II, IX and XII and molecular docking studies

**DOI:** 10.1080/14756366.2019.1666836

**Published:** 2019-09-20

**Authors:** Ciro Milite, Giorgio Amendola, Alessio Nocentini, Silvia Bua, Alessandra Cipriano, Elisabetta Barresi, Alessandra Feoli, Ettore Novellino, Federico Da Settimo, Claudiu T. Supuran, Sabrina Castellano, Sandro Cosconati, Sabrina Taliani

**Affiliations:** a Department of Pharmacy, Epigenetic Med Chem Lab, University of Salerno, Fisciano (SA), Italy;; b DiSTABiF, Università della Campania Luigi Vanvitelli, Caserta, Italy;; c NEUROFARBA Department, Sezione di Scienze Farmaceutiche e Nutraceutiche, Università degli Studi di Firenze, Sesto Fiorentino (Florence), Italy;; d PhD Program in Drug Discovery and Development, University of Salerno, Fisciano (SA), Italy;; e Department of Pharmacy, Universy of Pisa, Pisa, Italy;; f Department of Pharmacy, University Federico II of Naples, Naples, Italy

**Keywords:** Carbonic anhydrase inhibitors, benzimidazole-sulfonamides, reduced flexibility approach, isoform-selective inhibitors, molecular docking

## Abstract

Inhibition of Carbonic Anhydrases (CAs) has been clinically exploited for many decades for a variety of therapeutic applications. Within a research project aimed at developing novel classes of CA inhibitors (CAIs) with a proper selectivity for certain isoforms, a series of derivatives featuring the 2-substituted-benzimidazole-6-sulfonamide scaffold, conceived as frozen analogs of Schiff bases and secondary amines previously reported in the literature as CAIs, were investigated. Enzyme inhibition assays on physiologically relevant human CA I, II, IX and XII isoforms revealed a number of potent CAIs, showing promising selectivity profiles towards the transmembrane tumor-associated CA IX and XII enzymes. Computational studies were attained to clarify the structural determinants behind the activities and selectivity profiles of the novel inhibitors.

## Graphical Abstract

**Figure:**
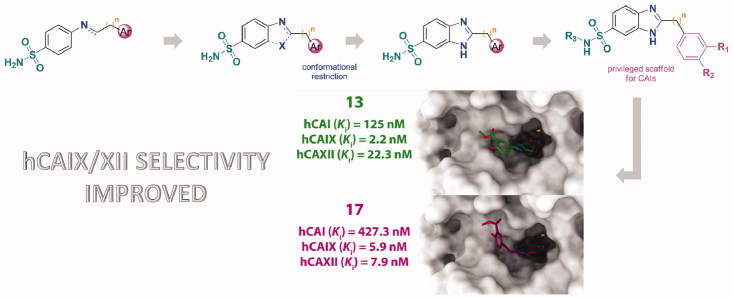

## Introduction

Carbonic anhydrases (CA) are a family of ubiquitary zinc metalloenzymes that catalyze the reversible reaction of hydration of CO_2_ to HCO_3_^−^ ^1^. This simple transformation plays a physiological regulatory role in a number of processes associated with pH control, ion transport, fluid secretion and several biosynthetic pathways^1^. Fifteen CA isoenzymes are encoded in humans and other primates, that differ for their subcellular localization, catalytic activity, and susceptibility to different classes of inhibitors^1^. Specifically, cytosolic (CA I, CA II, CA III, CA VII, and CA XIII), membrane-bound (CA IV, CA IX, CA XII and CA XIV), mitochondrial (CA VA and CA VB), and secreted in saliva (CA VI) enzymes were characterized^1^. Most of these CA isoforms represent interesting therapeutic targets, and their inhibition has been exploited clinically for many decades for a variety of applications in treating a multitude of diseases such as glaucoma, edema, epilepsy, obesity, neuropathic pain and other neurological disorders^2–4^. More recently, hCA IX and XII have been implicated in tumor progression/metastasis, and their selective inhibition could represent an additional opportunity for drug intervention against hypoxic cancers^5^.

**Chart 1.**
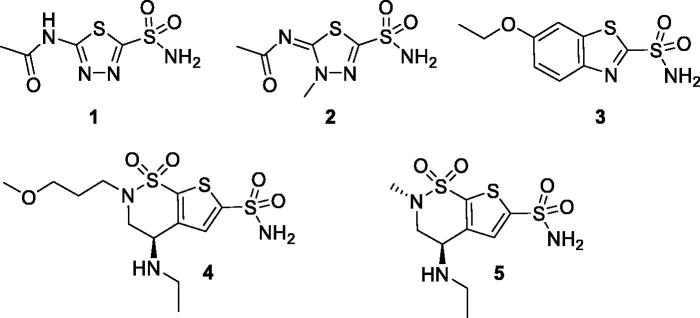
Structures of clinically used CAIs.

Sulfonamides and their bioisosteres (sulfamates, sulfamides) are known, powerful CA inhibitors (CAIs)^6^. Acetazolamide **1** (AAZ), methazolamide **2**, ethoxzolamide **3**, brinzolamide **4** and dorzolamide **5** are among the CAIs used in medicine mainly as diuretics and antiglaucoma agents (Chart 1)^6^.

Because of the ubiquity of CAs, the selectivity of the inhibitors for certain isoforms is a crucial issue to be reached in a drug development campaign in order to target a disease without relevant side effects^7^. In this respect, expanding the chemical space by the exploration of novel scaffolds may aid the development of novel classes of CAIs featuring improved pharmacological properties in terms of inhibition potency and isoform-selectivity.

**Figure 1.**
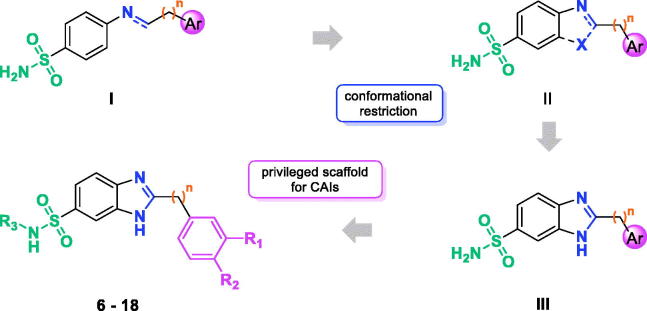
Flowchart of our frozen analog approach.

Among the several sulfonamide CAIs described, Schiff bases and secondary amines incorporating aromatic/heterocyclic sulfonamide moieties in their structure (compounds of type **I** in Figure 1) have been extensively investigated in recent years^8–11^. Comparing the activity of imines and their secondary amine counterparts, it is evident that the molecular flexibility markedly affects, positively or negatively, both activity and selectivity^8–11^.

Prompted by these outcomes and exploiting our experience in the application of the frozen analog approach^12^^,^^13^, we decided to further reduce the flexibility of the Schiff bases constraining the N=C imine bond into a ring (**II**, Figure 1). As a rigidifying building block, we decided to introduce the benzimidazole (**III**, Figure 1), a privileged structure extensively used in medicinal chemistry, which has been only scarcely explored for its potential in the development of CAIs^14^. As substituent in the position 2 of benzimidazole core, we selected phenols and benzoic acid derivatives. In fact, different studies showed that these scaffolds are effective CAIs, giving interactions with the enzyme that may not involve a direct interaction with the active site zing ion^15–17^. Herein, we report the synthesis of 2-substituted-benzimidazole-6-sulfonamides **6–18** and the related carboxamide **19** and their inhibitory activity toward four physiologically relevant enzymes, the cytosolic isoforms hCA I and II as well as the transmembrane tumor-associated ones hCA IX and XII.

## Materials and methods

### Chemistry

All chemicals were purchased from Sigma Aldrich Srl (Milan, Italy) or from Fluorochem Ltd. (Hadfield, UK) and were of the highest purity. All solvents were reagent grade and, when necessary, were purified and dried by standard methods. All reactions requiring anhydrous conditions were conducted under a positive atmosphere of nitrogen in oven-dried glassware. Standard syringe techniques were used for anhydrous addition of liquids. Reactions were routinely monitored by TLC performed on aluminum-backed silica gel plates (Merck DC, Alufolien Kieselgel 60 F_254_) with spots visualized by UV light (λ = 254, 365 nm) or using a KMnO_4_ alkaline solution. Solvents were removed using a rotary evaporator operating at a reduced pressure of ∼10 Torr. Organic solutions were dried over anhydrous Na_2_SO_4_. Chromatographic purification was done on an automated flash-chromatography system (IsoleraTM Dalton 2000, Biotage) using cartridges packed with KP-SIL, 60 Å (40–63 µm particle size). All microwave assisted reactions were conducted in a CEM Discover^®^ SP microwave synthesizer equipped with a vertically focused IR temperature sensor. Analytical high performance liquid chromatography (HPLC) was performed on a Shimadzu SPD 20 A UV/VIS detector (λ = 220 and 254 nm) using C-18 column Phenomenex Synergi Fusion - RP 80 A (75 × 4.60 mm; 4 µm) at 25 °C using a mobile phase A (water + 0.1% TFA) and B (ACN + 0.1% TFA) at a flow rate of 1 ml/min. ^1^H spectra were recorded at 400 MHz on a Bruker Ascend 400 spectrometer while ^13^C NMR spectra were obtained by distortionless enhancement by polarization transfer quaternary (DEPTQ) spectroscopy on the same spectrometer. Chemical shifts are reported in *δ* (ppm) relative to the internal reference tetramethylsilane (TMS). Due to the existence of tautomers, some ^1^H and ^13^C NMR signals could not be detected for some of the prepared benzimidazoles so only the distinct signals are reported. Low resolution mass spectra were recorded on a Finnigan LCQ DECA TermoQuest mass spectrometer in electrospray positive and negative ionization modes (ESI-MS). High resolution mass spectra were recorded on a Bruker solariX MRMS in electrospray positive ionization modes (ESI-FTMS). All tested compounds possessed a purity of at least 95% established by HPLC unless otherwise noted. Acids **27** and **28a** were commercially available, acid **28b** was obtained by previously reported procedure (see Supplementary Data).

#### 2-(4-Hydroxyphenyl)-1H-benzo[d]imidazole-6-sulfonamide (6)

*N*-(tert-butyl)-2-(4-hydroxyphenyl)-1*H*-benzo[*d*]imidazole-6-sulfonamide **26a** (174 mg, 0.504 mmol) was dissolved in 2 ml of a solution DCM/TFA (1:1) and the mixture was stirred for 18 h. The solvent was evaporated, and the resulting solid was crystallized with ethanol to give the title compound as a brown solid (110 mg, 75%). ^1^H NMR (400 MHz, DMSO-d_6_) *δ* 10.12 (s, 1H, exchangeable with D_2_O), 8.03 (d, *J* = 8.3 Hz, 2H), 7.99 (s, 1H), 7.72–7.64 (m, 2H), 7.28 (s, 2H, exchangeable with D_2_O), 6.95 (d, *J =* 8.3 Hz, 2H). ^13^C NMR (100 MHz, DMSO) *δ* 159.92, 154.19, 137.88, 128.67, 119.73, 115.85. HRMS (ESI): m/z [M + H]^+^ calcd for C_13_H_11_N_3_O_3_S + H^+^, 290.05939; found, 290.05938.

#### 2-(4-Hydroxybenzyl)-1H-benzo[d]imidazole-6-sulfonamide (7)

Compound **7** was obtained as a white solid (47 mg, 75%) by reaction of **31** (74 mg, 0.206 mmol) following the procedure described for **6**. ^1^H NMR (400 MHz, DMSO-d_6_) *δ* 9.42 (s, 1H, exchangeable with D_2_O), 8.04 (s, 1H), 7.79–7.73 (m, 2H), 7.39 (s, 2H, exchangeable with D_2_O), 7.16 (d, *J =* 8.0 Hz, 2H), 6.75 (d, *J =* 7.8 Hz, 2H), 4.26 (s, 2H). ^13^C NMR (100 MHz, DMSO) *δ* 156.71, 156.58, 139.31, 130.02, 125.27, 120.94, 115.54, 114.50, 112.54, 32.95. HRMS (ESI): m/z [M + H]^+^ calcd for C_14_H_13_N_3_O_3_S + H^+^, 304.07504; found, 304.07503.

#### 2-(4-Hydroxyphenethyl)-1H-benzo[d]imidazole-6-sulfonamide (8)

Compound **30a** (245 mg, 0.626 mmol) was dissolved in 80 ml of toluene, *p*-toluensulfonic acid (59 mg, 0.313 mmol) was added and the resulting mixture was heated at reflux for 6 h. Solvent was evaporated and the crude residue was taken up with a saturated solution of NaHCO_3_ (30 ml). The aqueous phase was extracted with EtOAc (3 × 20 ml) and the collected organic phases were washed with saturated solution of NaHCO_3_ (3 × 20 ml), brine (20 ml), anhydrified over Na_2_SO_4_, filtered and concentrated under reduced pressure. The title compound was obtained after crystallization with ethanol as a light brown solid (130 mg, 65%). ^1^H NMR (400 MHz, DMSO-d_6_) *δ* 9.15 (s, 1H, exchangeable with D_2_O), 7.93 (s, 1H), 7.63–7.58 (m, 2H), 7.21 (s, 2H, exchangeable with D_2_O), 7.01 (d, *J =* 8.1 Hz, 2H), 6.64 (d, *J =* 8.1 Hz, 2H), 3.09 (t, *J =* 7.5 Hz, 2H), 2.99 (t, *J =* 7.5 Hz, 2H). ^13^C NMR (100 MHz, DMSO) *δ* 155.56, 137.19, 130.80, 129.05, 119.01, 115.09, 32.44, 30.88. HRMS (ESI): m/z [M + H]^+^ calcd for C_15_H_15_N_3_O_3_S + H^+^, 318.09069; found, 318.09066

#### 2-Phenyl-1H-benzo[d]imidazole-6-sulfonamide (9)

Compound **9** was obtained as a light brown solid (49 mg, 75%) by reaction of **26b** (80 mg, 0.24 mmol) following the procedure described for **6**. ^1^H NMR (400 MHz, DMSO-d_6_) *δ* 8.25–8.16 (m, 2H), 8.09 (d, *J =* 1.8 Hz, 1H), 7.82 (d, *J =* 8.7 Hz, 1H), 7.77 (dd, *J =* 8.5, 1.8 Hz, 1H), 7.68–7.59 (m, 3H), 7.37 (s, 2H, exchangeable with D_2_O). ^13^C NMR (100 MHz, DMSO) *δ* 153.36, 138.92, 131.25, 129.22, 128.00, 127.07, 120.64. HRMS (ESI): m/z [M + H]^+^ calcd for C_13_H_11_N_3_O_2_S + H^+^, 274.06447; found, 274.06445.

#### Methyl 4-(6-sulfamoyl-1H-benzo[d]imidazol-2-yl)benzoate (10)

Compound **10** was obtained as a light brown solid (49 mg, 70%) by reaction of **26c** (82 mg, 0.212 mmol) following the procedure described for **6**. ^1^H NMR (400 MHz, DMSO-d_6_) 8.38–8.32 (m, 2H), 8.20–8.13 (m, 2H, 1H exchangeable with D_2_O), 8.00–7.85 (m, 1H), 7.78–7.68 (m, 1H), 7.33 (m, 2H, 1H exchangeable with D_2_O), 3.91 (s, 3H). ^13^C NMR (100 MHz, DMSO) *δ* 165.71, 152.86, 152.43, 145.64, 142.87, 138.78, 138.31, 137.06, 134.23, 133.61, 130.84, 129.87, 126.96, 120.73, 119.74, 119.35, 117.09, 111.97, 109.84, 52.33. HRMS (ESI): *m/z* [M + H]^+^ calcd for C_15_H_13_N_3_O_4_S + H^+^, 332.06995; found, 332.06993.

#### 4-(6-Sulfamoyl-1H-benzo[d]imidazol-2-yl)benzoic acid (11)

To a stirred solution of compound **10** (250 mg, 0.645 mmol) in 1.5 ml of THF was added a water solution (1.5 ml) of LiOH (62 mg, 2.58 mmol). The reaction mixture was stirred at room temperature for 3 h and then concentrated under vacuum. The aqueous phase was washed with CHCl_3_ then acidified with 3N HCl until a white precipitate formed. After filtration, the title compound was obtained as white solid (200 mg, 83%).^1^H NMR (400 MHz, DMSO-d_6_) *δ* 8.32 (d, *J =* 8.2 Hz, 2H), 8.16–8.11 (m, 3H), 7.84–7.68 (m, 2H), 7.32 (s, 2H, exchangeable with D_2_O). ^13^C NMR (100 MHz, DMSO) *δ* 166.76, 133.24, 132.14, 129.99, 126.83. HRMS (ESI): *m/z* [M + H]^+^ calcd for C_14_H_11_N_3_O_4_S + H^+^, 318.05430; found, 318.05429.

#### 2-(3,4-Dihydroxyphenyl)-1H-benzo[d]imidazole-6-sulfonamide (12)

Compound **12** was obtained as a light brown solid (105 mg, 73%) by reaction of **26d** (170 mg, 0.470 mmol) following the procedure described for **6**. ^1^H NMR (400 MHz, DMSO-d_6_) *δ* 9.54 (s, 1H, exchangeable with D_2_O), 9.31 (s, 1H, exchangeable with D_2_O), 8.02, 7.90 (2 s, 1H), 7.73–7.55 (m, 3H), 7.48 (dd, *J =* 8.4, 1.7 Hz, 1H), 7.31–7.17 (m, 2H, exchangeable with D_2_O), 6.89 (d, *J =* 8.5 Hz, 1H). ^13^C NMR (100 MHz, DMSO) *δ* 148.09, 145.63, 143.10, 137.50, 120.73, 119.53, 119.16, 118.58, 118.18, 116.13, 115.80, 114.26, 110.98, 109.09. HRMS (ESI): *m/z* [M + H]^+^ calcd for C_13_H_11_N_3_O_4_S + H^+^, 306.05430; found, 306.05431.

#### Methyl 2-hydroxy-5-(6-sulfamoyl-1H-benzo[d]imidazol-2-yl)benzoate (13)

Compound **13** was obtained as a light brown solid (38 mg, 75%) by reaction of **26e** (60 mg, 0.149 mmol) following the procedure described for **6**. ^1^H NMR (400 MHz, DMSO-d_6_) *δ* 10.94 (s, 1H, exchangeable with D_2_O), 8.65 (d, *J =* 2.3 Hz, 1H), 8.32 (dd, *J =* 8.8, 2.2 Hz, 1H), 8.04 (s, 1H), 7.80–7.70 (m, 2H), 7.35 (s, 2H, exchangeable with D_2_O), 7.23 (dd, *J =* 8.8, 2.0 Hz, 1H), 3.97 (s, 3H). ^13^C NMR (100 MHz, DMSO) *δ* 168.12, 161.55, 152.68, 138.60, 133.57, 129.30, 120.36, 118.50, 114.47, 52.64. HRMS (ESI): *m/z* [M + H]^+^ calcd for C_15_H_13_N_3_O_5_S + H^+^, 348.06487; found, 348.06486.

#### 2-hydroxy-5-(6-sulfamoyl-1H-benzo[d]imidazol-2-yl)benzoic acid (14)

Compound **14** was obtained as white solid (120 mg, 83%) by reaction of **13** (150 mg, 0.43 mmol) following the procedure described for **11**. ^1^H NMR (400 MHz, DMSO-d_6_) *δ* 8.68 (d, *J =* 2.4 Hz, 1H), 8.32 (dd, *J =* 8.7, 2.5 Hz, 1H), 8.02 (s, 1H), 7.75–7.67 (m, 2H), 7.30 (s, 2H, exchangeable with D_2_O), 7.17 (dd, *J =* 8.8, 2.2 Hz, 1H). ^13^C NMR (100 MHz, DMSO) *δ* 171.28, 163.06, 153.18, 138.07, 133.57, 129.08, 119.88, 118.11, 114.04. HRMS (ESI): *m/z* [M + H]^+^ calcd for C_14_H_11_N_3_O_5_S + H^+^, 334.04922; found, 334,04922.

#### Methyl 5-(6-(N-ethylsulfamoyl)-1H-benzo[d]imidazol-2-yl)-2-hydroxybenzoate (15)

Compound **15** was obtained as a light brown solid (235 mg, 71%) by reaction of **20b** (188 mg, 0.88 mmol) and methyl 5-formylsalicylate (160 mg, 0.88 mmol) following the procedure described for **6**.

^1^H NMR (400 MHz, DMSO-d_6_) *δ* 10.85 (s, 1H, exchangeable with D_2_O), 8.65 (d, *J =* 2.4 Hz, 1H), 8.32 (dd, *J =* 8.7, 2.4 Hz, 1H), 7.99–7.96 (m, 1H), 7.79–7.68 (m, 1H), 7.68–7.59 (m, 1H), 7.47 (t, *J =* 5.7 Hz, 1H, exchangeable with D_2_O), 7.20 (d, *J =* 8.7 Hz, 1H), 3.97 (s, 3H), 2.83 – 2.72 (m, 2H), 0.96 (t, *J =* 7.2 Hz, 3H). ^13^C NMR (100 MHz, DMSO) *δ* 168.31, 161.28, 153.22, 133.98, 133.47, 128.91, 120.80, 120.49, 118.38, 114.19, 52.62, 37.55, 14.65. HRMS (ESI): *m/z* [M + H]^+^ calcd for C_17_H_17_N_3_O_5_S + H^+^, 376.09617; found, 376.09620.

#### 5-(6-(N-ethylsulfamoyl)-1H-benzo[d]imidazol-2-yl)-2-hydroxybenzoic acid (16)

Compound **16** was obtained as a white solid (128 mg, 89%) by reaction of **15** (150 mg, 0.40 mmol) following the procedure described for **11**. ^1^H NMR (400 MHz, DMSO-d_6_) 8.71 (d, *J =* 2.3 Hz, 1H), 8.36 (dd, *J =* 8.8, 2.3 Hz, 1H), 8.01 (s, 1H), 7.79 (d, *J =* 8.5 Hz, 1H), 7.69 (dd, *J =* 8.5, 1.8 Hz, 1H), 7.55 (t, *J =* 5.9 Hz, 1H, exchangeable with D_2_O), 7.21 (d, *J =* 8.8 Hz, 1H), 2.85–2.72 (m, 2H), 0.97 (t, *J =* 7.3 Hz, 3H). ^13^C NMR (100 MHz, DMSO) *δ* 171.17, 163.22, 152.94, 134.66, 133.87, 129.51, 121.09, 118.27, 114.01, 37.56, 14.66. HRMS (ESI): *m/z* [M + H]^+^ calcd for C_16_H_15_N_3_O_5_S + H^+^, 362.08052; found, 362.08061.

#### Methyl 2-hydroxy-5-(2-(6-sulfamoyl-1H-benzo[d]imidazol-2-yl)ethyl)benzoate (17)

Compound **17** was obtained as a white solid (285 mg, 60%) by reaction of **30b** (570 mg, 1.27 mmol) following the procedure described for **8**. ^1^H NMR (400 MHz, DMSO-d_6_) *δ* 10.32 (s, 1H, exchangeable with D_2_O) , 7.98, 7.87 (2 s, 1H), 7.68–7.54 (m, 3H), 7.39 (dd, *J =* 8.5, 2.4 Hz, 1H), 7.25–7.19 (m, 2H, exchangeable with D_2_O), 6.89 (d, *J =* 8.4 Hz, 1H), 3.85 (s, 3H), 3.15–3.04 (m, 4H). ^13^C NMR (100 MHz, DMSO) *δ* 169.13, 158.41, 157.48, 156.87, 145.32, 142.42, 137.19, 136.26, 135.75, 131.61, 129.27, 119.29, 118.73, 118.11, 117.36, 116.10, 112.64, 110.93, 109.07, 52.34, 32.08, 30.52. HRMS (ESI): *m/z* [M + H]^+^ calcd for C_17_H_17_N_3_O_5_S + H^+^, 376.09617; found, 376.09613.

#### 2-Hydroxy-5-(2-(6-sulfamoyl-1H-benzo[d]imidazol-2-yl)ethyl)benzoic acid (18)

Compound **18** was obtained as a white solid (135 mg, 93%) by reaction of **17** (150 mg, 0.400 mmol) following the procedure described for **11**. ^1^H NMR (400 MHz, DMSO-d_6_) *δ* 8.14 (s, 1H), 7.91–7.89 (m, 2H), 7.70 (d, *J =* 2.4 Hz, 1H), 7.51 (s, 2H, exchangeable with D_2_O), 7.47–7.37 (m, 1H), 6.89 (d, *J =* 8.5 Hz, 1H), 3.39 (t, *J =* 7.8 Hz, 2H), 3.18 (t, *J =* 7.8 Hz, 2H). ^13^C NMR (100 MHz, DMSO) *δ* 171.64, 159.74, 156.30, 140.72, 135.63, 129.98, 129.65, 122.28, 117.32, 114.47, 112.82, 112.00, 31.19, 28.83.HRMS (ESI): *m/z* [M + H]^+^ calcd for C_16_H_15_N_3_O_5_S + H^+^, 362.08052; found, 362.08052.

#### 5-(6-Carbamoyl-1H-benzo[d]imidazol-2-yl)-2-hydroxybenzoic acid (19)

Compound **35** (150 mg, 0.408 mmol) was dissolved in 2 ml of a solution DCM/TFA (1:1) and the mixture was stirred for 24 h. The solvent was evaporated, and the resulting solid was taken up with 2 ml of THF. To the resulting mixture, a water solution (2 ml) of LiOH (62 mg, 2.58 mmol) was added, and the reaction mixture was stirred at room temperature for 4 h. The reaction was concentrated under vacuum, the aqueous phase was washed with CHCl_3_ then acidified with 3N HCl until a white precipitate formed that was recovered by filtration. Compound **19** was obtained as white solid (110 mg, 91%) after recrystallization from ethanol ^1^H NMR (400 MHz, DMSO-d_6_) *δ* 8.72 (d, *J =* 2.4 Hz, 1H), 8.36–8.26 (m, 1H), 8.17 (s, 1H), 8.09 (s, 1H, exchangeable with D_2_O), 7.88 (d, *J =* 8.4 Hz, 1H), 7.71–7.65 (m, 1H), 7.38 (s, 1H, exchangeable with D_2_O), 7.19 (d, *J =* 8.7 Hz, 1H). ^13^C NMR (100 MHz, DMSO) *δ* 171.54, 168.31, 164.41, 152.09, 134.20, 130.25, 130.08, 123.63, 118.84, 115.01. HRMS (ESI): *m/z* [M + H]^+^ calcd for C_15_H_11_N_3_O_4_ + H^+^, 298.08223; found, 298.08230.

#### 3,4-Diamino-N-(tert-butyl)benzenesulfonamide (20a)

To a stirred suspension of **24a** (1.65 g, 6.04 mmol) in 250 ml of MeOH, ammonium formate (7.61 g, 120.74 mmol) and palladium on carbon 10% wt. (160 mg) were added. The resulting mixture was heated at reflux for 4 h. After cooling, the mixture was filtered, and the solvent evaporated under reduced pressure. The crude material was taken up with 100 ml of water and extracted with EtOAc (3 × 60 ml). The combined organic phases were washed with brine, dried over Na_2_SO_4_, filtered and evaporated. The product, obtained as a light brown solid (1.30 g, 88%), was used for the next step without further purification. ^1^H NMR (400 MHz, DMSO-d_6_) *δ* 6.96 (d, *J =* 2.1 Hz, 1H), 6.90–6.84 (m, 2H, 1H exchangeable with D_2_O), 6.53 (d, *J =* 8.2 Hz, 1H), 5.13 (brs, 2H, exchangeable with D_2_O), 4.79 (brs, 2H, exchangeable with D_2_O), 1.08 (s, 9H). ESI *m/z*: 244 [M + H]^+^.

#### 3,4-Diamino-N-ethylbenzenesulfonamide (20b)

Compound **20b** was obtained as a light brown solid (910 mg, 94%) by reaction of **24b** (1.10 g, 4.48 mmol) following the procedure described for **20a**. ^1^H NMR (400 MHz, DMSO-d_6_) *δ* 6.94–6.88 (m, 2H, 1H exchangeable with D_2_O), 6.81 (dd, *J =* 8.0, 2.1 Hz, 1H), 6.53 (d, *J =* 8.0 Hz, 1H), 5.18 (brs, 2H, exchangeable with D_2_O), 4.82 (brs, 2H, exchangeable with D_2_O), 2.75–2.62 (m, 2H), 0.94 (t, *J =* 7.2 Hz, 3H). ESI *m/z*: 216 [M + H]^+^.

#### Ethyl 2-((2-nitrophenyl)amino)-2-oxoacetate (22)

To a solution of 2-nitroaniline (2.00 g, 14.48 mmol) in Et_2_O (100 ml), ethyl chlorooxoacetate (2.17 g, 1.78 ml, 15.93 mmol) was added portionwise with continuous stirring. Once the addition was complete, the resulting yellow suspension was stirred for 18 h at room temperature and then concentrated under vacuo. The crude residue was dissolved in EtOAc (100 ml), washed with saturated NaHCO_3_ (3 × 30 ml) and with brine (30 ml). The organic phase was dried over anhydrous Na_2_SO_4_, and evaporated to dryness, giving the desired product as a yellow solid (3.38 g, 98%). ^1^H NMR (400 MHz, DMSO-d_6_) *δ* 11.38 (s, 1H), 8.17–8.05 (m, 2H), 7.85–7.76 (m, 1H), 7.52–7.41 (m, 1H), 4.34 (q, *J =* 7.0 Hz, 2H), 1.33 (t, *J =* 7.0 Hz, 3H). ESI *m/z*: 239 [M + H]^+^.

#### 4-Amino-3-nitrobenzenesulfonyl chloride (23)

A solution of ethyl 2-(2-nitrophenylamino)-2-oxoacetate **22** (2.00 g, 8.40 mmol) in 4.5 ml of chlorosulfonic acid was heated at 80 °C for 3 h. The red mixture was poured slowly into ice − water (150 ml) and stirred for 30 min. The product was extracted from the aqueous solution using Et_2_O (3 × 30 ml). The combined organic phases were washed with brine (10 ml), dried (Na_2_SO_4_), filtered, and concentrated *in vacuo* to give the title compound as a brown solid which was immediately used for the next reaction without purification. ^1^H NMR (400 MHz, DMSO-d_6_) *δ* 8.16 (d, *J =* 2.0 Hz, 1H), 7.56 (dd, *J =* 8.8, 2.0 Hz, 1H), 6.96 (d, *J =* 8.8 Hz, 1H).

#### 4-Amino-N-(tert-butyl)-3-nitrobenzenesulfonamide (24a)

To a stirred solution at 0 °C of crude **23** (1.04 g, 4.39 mmol) in dry THF (25 ml) was added dropwise, under nitrogen atmosphere, *tert*-butylamine (1.85 ml, 17.56 mmol). The reaction was allowed to reach room temperature and was stirred for 18 h. The solvent was removed at reduced pressure and the residue was taken up with 50 ml of water and extracted with EtOAc (3 × 20 ml). The combined organic phases were washed with brine, dried over Na_2_SO_4_, filtered and evaporated under reduced pressure. Purification by silica gel chromatography (DCM/MeOH) yield pure **24a** (0.96 g, 80%) as a light yellow solid. ^1^H NMR (400 MHz, DMSO-d_6_) *δ* 8.38 (d, *J =* 2.2 Hz, 1H), 7.95 (s, 2H, exchangeable with D_2_O), 7.70 (dd, *J =* 9.0, 2.2 Hz, 1H), 7.44 (s, 1H, exchangeable with D_2_O), 7.11 (d, *J =* 9.0 Hz, 1H), 1.10 (s, 9H). ESI *m/z*: 274 [M + H]^+^.

#### 4-Amino-N-ethyl-3-nitrobenzenesulfonamide (24b)

Compound **24b** was obtained as a yellow solid (840 mg, 78%) by reaction of **23** (1.04 g, 4.39 mmol) with a 2 M THF solution of ethylamine (8.78 ml, 17.56 mmol) following the procedure described for **24a**. ^1^H NMR (400 MHz, DMSO-d_6_) *δ* 8.36 (d, J = 1.6 Hz, 1H), 8.01 (s, 2H, exchangeable with D_2_O), 7.69 (dd, J = 9.0, 1.6 Hz, 1H), 7.54–7.44 (m, 1H, exchangeable with D_2_O), 7.15 (d, J = 9.0 Hz, 1H), 2.82 – 2.72 (m, 2H), 0.99 (t, J = 7.2 Hz, 3H). ESI *m/z*: 246 [M + H]^+^.

#### N-(tert-butyl)-2-(4-hydroxyphenyl)-1H-benzo[d]imidazole-6-sulfonamide (26a)

To a stirred solution of **20a** (150 mg, 0.62 mmol) in dry DMF (7 ml), 4-hydroxybenzaldehyde (75 mg, 0.61 mmol) and Na_2_S_2_O_5_ (0.165 g, 0.793 mmol) were added. The resulting mixture was stirred at 80 °C for 18 h. After cooling at room temperature, water was added. The brown precipitate formed was recovered by filtration and was washed several times with water and 1N HCl. After recrystallization from EtOH, compound **26a** was obtained as light brown solid (160 mg, 72%). ^1^H NMR (400 MHz, DMSO-d_6_) *δ* 10.07 (s, 1H, exchangeable with D_2_O), 8.03 (d, *J =* 8.5 Hz, 2H), 8.01–7.96 (m, 1H), 7.69–7.61 (m, 2H), 7.43 (s, 1H, exchangeable with D_2_O), 6.94 (d, *J =* 8.5 Hz, 2H), 1.09 (s, 9H).ESI *m/z*: 346 [M + H]^+^.

#### N-(tert-butyl)-2-phenyl-1H-benzo[d]imidazole-6-sulfonamide (26b)

Compound **26b** was obtained as a light brown solid (204 mg, 65%) by reaction of **20a** (233 mg, 0.954 mmol) and benzaldehyde (97 µL, 0.954 mmol) following the procedure described for **26a**. ^1^H NMR (400 MHz, DMSO-d_6_) *δ* 8.23–8.15 (m, 2H), 8.11–8.04 (m, 1H), 7.85–7.72 (m, 2H), 7.68–7.58 (m, 3H), 7.54 (s, 1H, exchangeable with D_2_O), 1.09 (s, 9H).ESI *m/z*: 330 [M + H]^+^.

#### Methyl 4-(6-(N-(tert-butyl)sulfamoyl)-1H-benzo[d]imidazol-2-yl)benzoate (26c)

Compound **26c** was obtained as a light brown solid (190 mg, 83%) by reaction of **20a** (144 mg, 0.590 mmol) and methyl 4-formylbenzoate (97 mg, 0.590 mmol) following the procedure described for **26a**. ^1^H NMR (400 MHz, DMSO-d_6_) *δ* 8.35 (d, *J =* 8.0 Hz, 2H), 8.17 (d, *J =* 8.0 Hz, 2H), 8.09 (s, 1H), 7.83–7.70 (m, 2H), 7.51 (s, 1H, exchangeable with D_2_O), 3.91 (s, 3H), 1.10 (s, 9H). ESI *m/z*: 388 [M + H]^+^.

#### N-(tert-butyl)-2-(3,4-dihydroxyphenyl)-1H-benzo[d]imidazole-6-sulfonamide (26d)

Compound **26d** was obtained as a light brown solid (290 mg, 65%) by reaction of **20a** (300 mg, 1.23 mmol) and 3,4-dihydroxybenzaldehyde (170 mg, 1.23 mmol) following the procedure described for **26a**. ^1^H NMR (400 MHz, DMSO-d_6_) *δ* 9.42 (brs, 2H, exchangeable with D_2_O), 8.02, 7.90 (2 s, 1H), 7.73–7.58 (m, 3H), 7.49 (dd, *J =* 8.2, 2.1 Hz, 1H), 7.42 (s, 1H, exchangeable with D_2_O), 6.90 (d, *J =* 8.3 Hz, 1H), 1.09 (s, 9H).ESI *m/z*: 362 [M + H]^+^.

#### Methyl 5-(6-(N-(tert-butyl)sulfamoyl)-1H-benzo[d]imidazol-2-yl)-2-hydroxybenzoate (26e)

Compound **26e** was obtained as a light brown solid (222 mg, 71%) by reaction of **20a** (188 mg, 0.773 mmol) and methyl 5-formylsalicylate (139 mg, 0.773 mmol) following the procedure described for **26a**. ^1^H NMR (400 MHz, DMSO-d_6_) *δ* 8.65 (d, *J =* 2.3 Hz, 1H), 8.32 (dd, *J =* 8.7, 2.3 Hz, 1H), 8.01 (s, 1H), 7.76–7.64 (m, 2H), 7.46 (s, 1H, exchangeable with D_2_O), 7.19 (d, *J =* 8.7 Hz, 1H), 3.97 (s, 3H), 1.09 (s, 9H). ESI *m/z*: 404 [M + H]^+^.

#### N-(2-amino-4-(N-(tert-butyl)sulfamoyl)phenyl)-2-(4-hydroxyphenyl)acetamide and N-(2-amino-5-(N-(tert-butyl)sulfamoyl)phenyl)-2-(4-hydroxyphenyl)acetamide (29)

To a stirred solution of **20a** (354 mg, 1.45 mmol) and 4-hydroxyphenylacetic acid **27** (200 mg, 1.31 mmol) in dry DMF (26 ml) were added, EDC hydrochloride (427 mg, 2.23 mmol), HOBt (341 mg, 2.23 mmol) and 4-methylmorpholine (4.84 ml, 4.45 mmol). The reaction was stirred at room temperature for 18 h. Water (80 ml) was added the resulting mixture was extracted with EtOAc (3 × 40 ml). The combined organic phases were washed with brine (3 × 50 ml), dried over Na_2_SO_4_, filtered and evaporated under reduced pressure. Purification by silica gel chromatography (DCM/MeOH) yield an isomeric mixture as a white solid (0.360 g, 73%) that was used for the next step without separation. ESI *m/z*: 378 [M + H]^+^.

#### N-(2-amino-4-(N-(tert-butyl)sulfamoyl)phenyl)-3-(4-hydroxyphenyl)propanamide and N-(2-amino-5-(N-(tert-butyl)sulfamoyl)phenyl)-3-(4-hydroxyphenyl)propanamide (30a)

Compounds **30a** was obtained as a light yellow solid (300 mg, 64%) by reaction of **20a** (323 mg, 1.33 mmol) with 3-(4-hydroxyphenyl)propanoic acid **28a** (200 mg, 1.2 mmol) following the procedure described for **29**. ESI *m/z*: 392 [M + H]^+^.

#### Methyl 5-(3-((2-amino-4-(N-(tert-butyl)sulfamoyl)phenyl)amino)-3-oxopropyl)-2-hydroxybenzoate and methyl 5-(3-((2-amino-5-(N-(tert-butyl)sulfamoyl)phenyl)amino)-3-oxopropyl)-2-hydroxybenzoate (30b)

Compounds **30b** was obtained as a light yellow solid (90 mg, 90%) by reaction of **20a** (60 mg, 0.245 mmol) with 3-(4-hydroxy-3-(methoxycarbonyl)phenyl)propanoic acid **28b** (50 mg, 0.223 mmol) following the procedure described for **29**. ESI *m/z*: 450 [M + H]^+^.

#### N-(tert-butyl)-2-(4-hydroxybenzyl)-1H-benzo[d]imidazole-6-sulfonamide (31)

In a 10 ml CEM pressure vessel equipped with a stirrer bar, compounds **29** (50 mg, 0.132 mmol) were dissolved in 1.3 ml of acetic acid. The microwave vial was sealed and heated in a CEM Discover microwave synthesizer to 80 °C for 30 min. After cooling to room temperature, the reaction mixture was taken up with solution of NaHCO_3_ (60 ml) and the aqueous phase was extracted with EtOAc (3 × 20 ml). The collected organic phases were washed with water (3 × 20 ml), NaHCO_3_ solution (3 × 20 ml), brine (20 ml), dried over Na_2_SO_4_ and filtered. The solvent was removed under reduced pressure and the resulting crude material was purified by silica gel chromatography (DCM/EtOAc) to give compound **31** a light brown solid (35 mg, 74%). ^1^H NMR (400 MHz, DMSO-d_6_) *δ* 9.31 (s, 1H, exchangeable with D_2_O), 7.93 (s, 1H), 7.64–7.54 (m, 2H), 7.39 (s, 1H, exchangeable with D_2_O), 7.14 (d, *J =* 8.0 Hz, 2H), 6.71 (d, *J =* 8.0 Hz, 2H), 4.09 (s, 2H), 1.06 (s, 9H). ESI *m/z*: 360 [M + H]^+^.

#### N-(tert-butyl)-3,4-dinitrobenzamide (33)

A solution of 3,4-dinitrobenzoic acid **32** (1.00 g, 4.71 mmol) in thionyl chloride (15 ml) was refluxed for 2 h under a nitrogen atmosphere. After the solution was cooled at room temperature, the excess thionyl chloride was removed at reduced pressure and the crude material dried under vacuum. To the residue, dissolved in dry THF (20 ml) and cooled to 0 °C, was added dropwise a mixture of *tert*-butylamine (525 µL, 5.00 mmol) and triethylamine (695 µL, 5.00 mmol) in dry THF (5 ml). The mixture was stirred at room temperature for 18 h, filtered, and evaporated. The crude residue was dissolved in DCM (20 ml), washed with 1N HCl, saturated NaHCO_3_, and water, dried, and concentrated in vacuum. Recrystallization from EtOH yielded compound **33** (1.05 g, 84%) as a light yellow solid. ^1^H NMR (400 MHz, DMSO-d_6_) *δ* 8.57 (s, 1H), 8.35–8.29 (m, 2H), 1.40 (s, 9H). ESI *m/z*: 268 [M + H]^+^.

#### 3,4-Diamino-N-(tert-butyl)benzamide (34)

Compound **34** was obtained as a light brown solid (729 mg, 94%) by reaction of **33** (1.00 g, 3.74 mmol) following the procedure described for **20a**. ^1^H NMR (400 MHz, DMSO-d_6_) *δ* 7.05 (s, 1H, exchangeable with D_2_O), 6.98 (d, *J =* 2.0 Hz, 1H), 6.90 (dd, *J =* 8.1, 2.1 Hz, 1H), 6.45 (d, *J =* 8.0 Hz, 1H), 4.86 (s, 2H, exchangeable with D_2_O), 4.48 (s, 2H, exchangeable with D_2_O), 1.33 (s, 9H). ESI *m/z*: 208 [M + H]^+^.

#### Methyl 5-(6-(tert-butylcarbamoyl)-1H-benzo[d]imidazol-2-yl)-2-hydroxybenzoate (35)

Compound **35** was obtained as a light brown solid (304 mg, 72%) by reaction of **34** (240 mg, 1.15 mmol) and **25e** (208 mg, 1.15 mmol) following the procedure described for **6**. ^1^H NMR (400 MHz, DMSO-d_6_) *δ* 11.04 (s, 1H, exchangeable with D_2_O), 8.66 (d, *J =* 2.2 Hz, 1H), 8.30 (dd, *J =* 8.8, 2.4 Hz, 1H), 8.09 (s, 1H), 7.90–7.78 (m, 2H, 1H exchangeable with D_2_O), 7.68 (d, *J =* 8.4 Hz, 1H), 7.26 (d, *J =* 8.7 Hz, 1H), 3.97 (s, 3H), 1.42 (s, 9H). ESI *m/z*: 368 [M + H]^+^.

### Enzyme activity assays

An Applied Photophysics stopped-flow instrument has been used for assaying the CA-catalysed CO_2_ hydration activity^18^. Phenol red (at a concentration of 0.2 mM) has been used as indicator, working at the absorbance maximum of 557 nm, with 20 mM Hepes (pH 7.5) as buffer, and 20 mM Na_2_SO_4_ (for maintaining constant the ionic strength), following the initial rates of the CA-catalysed CO_2_ hydration reaction for a period of 10–100 s. CO_2_ concentrations ranged from 1.7 to 17 mM for the determining inhibition constants. For each inhibitor, at least six traces of the initial 5–10% of the reaction have been used for measuring the initial velocity. Uncatalyzed rates were determined in the same manner and subtracted from the total observed rates. Stock solutions of inhibitor (0.1 mM) were prepared in buffer with a maximum 3% DMSO, and dilutions up to 0.01 nM were done with the assay buffer. Inhibitor and enzyme solutions were preincubated for 15 min at room temperature prior to assay, in order to allow for the formation of the E–I complex. The inhibition constants were obtained by nonlinear least-squares methods using PRISM 3 and the Cheng-Prusoff equation, as reported earlier, and represent the mean from at least three different determinations^19–26^. All CA isoforms were recombinant ones obtained in-house as reported earlier^27–29^.

### Molecular modeling methods

The latest version of the AD4 docking software (version 4.2)^30^ together with its GUI AutoDockTools (ADT) and the AutoDock4(Zn) force field^31^, were employed. The hCA IX X-ray structure used for the experiment had the PDB code 5FL4^32^. The protein structure was prepared for the docking using the Protein Preparation Wizard of the Maestro suite^33^ that adds bond orders, adds hydrogen atoms, deletes water molecules and produces the appropriate protonation states. The co-crystal ligand of 5FL4 was separated from the cognate protein. The 2 D Sketcher tool of Maestro was used to build compounds **13**, **14** and **17**. For the three ligands, the protonation and tautomeric state, as well as their geometry, were optimized through LigPrep, part of the same suite. Through Maestro, the X-ray structures of hCA I (PDB 6F3B)^34^, hCA II (PDB 3K34)^35^, and hCA XII (PDB 5MSA)^36^, were downloaded and superimposed on the structure of hCA IX. The ligands were translated in the AD4 specific file format (PDBQT) using the python scripts prepare_ligand4.py and prepare_receptor4.py, part of ADT, applying the standard settings. Following the AutoDock4(Zn) force field protocol^37^, to add the tetrahedral zinc pseudo atoms to the receptor PDBQT the script zinc_pseudo.py, part of the material provided with the force field, was employed. The docking area was centered on the active site. The zinc-specific non bonded pairwise potentials were included in the creation of the grid parameter file. A set of grids of 60 Å × 40 Å × 50 Å with 0.375 Å spacing was calculated considering the docking area for all the ligands atom types employing AutoGrid4. For every ligand, 200 independent docking simulations were achieved. Each docking calculation comprised 20 million energy evaluations employing the Lamarckian genetic algorithm local search (GALS) method. This latter assesses a population of viable docking solutions and propagates the best individuals from each generation into the following generation of feasible solutions. A low-frequency local search according to the method of Solis and Wets was applied to every docking attempt to guarantee that the final solution represented a local minimum. All dockings were performed with a population size of 250, and 300 iterations of Solis and Wets local search were applied with a probability of 0.06. A rate of mutation of 0.02 and a crossover rate of 0.8 were used to produce new docking attempts for following generations, and the best individual from each generation was propagated over the following generation. The docking results from every (200) independent docking calculation were clustered based on the of root-mean-square deviation (rmsd) (solutions differing by less than 2.0 Å) between the Cartesian coordinates of the atoms and were ranked on the basis of free energy of binding (ΔGAD4).

## Results and discussion

### Chemistry

**Scheme 1.**
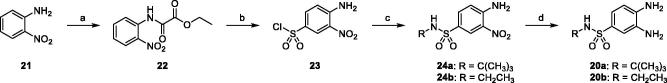
(a) Ethyl chlorooxoacetate, Et_2_O, r.t. 18 h (98%); (b) ClSO_3_H, 80 °C, 3 h (77%); c) *tert*-butylamine or 2M THF solution of ethylamine, THF, 0 °C to r.t., 18 h (80%); (d) ammonium formate, Pd/C 10%, MeOH, reflux, 4 h (88–94%).

The primary (**6–14** and **17–18**) and the *N*-ethyl-2-substituted-1*H*-benzo[*d*]imidazole-6-sulfonamides (**15** and **16**) were synthesized starting from 3,4-diaminobenzenesulfonamides **20a** and **20b**, respectively, obtained following the procedure outlined in Scheme 1.

The amino group of 2-nitroaniline **21** was protected by acylation with ethyl chlorooxoacetate in diethyl ether. Reaction of the resulting ethyl 2-(2-nitrophenylamino)-2-oxoacetate **22** with chlorosulfonic acid at 80 °C, followed by aqueous workup, yielded the unprotected sulfonyl chloride derivative **23**, which reacted with *tert-*butyl- or ethyl-amine to afford the *N*-substituted-4-amino-3-nitrobenzenesulfonamides **24a** and **24b**, respectively. Catalytic hydrogenation with ammonium formate and palladium catalyst converted the nitro derivatives into the corresponding amino derivatives **20a** and **20b**.

**Scheme 2.**
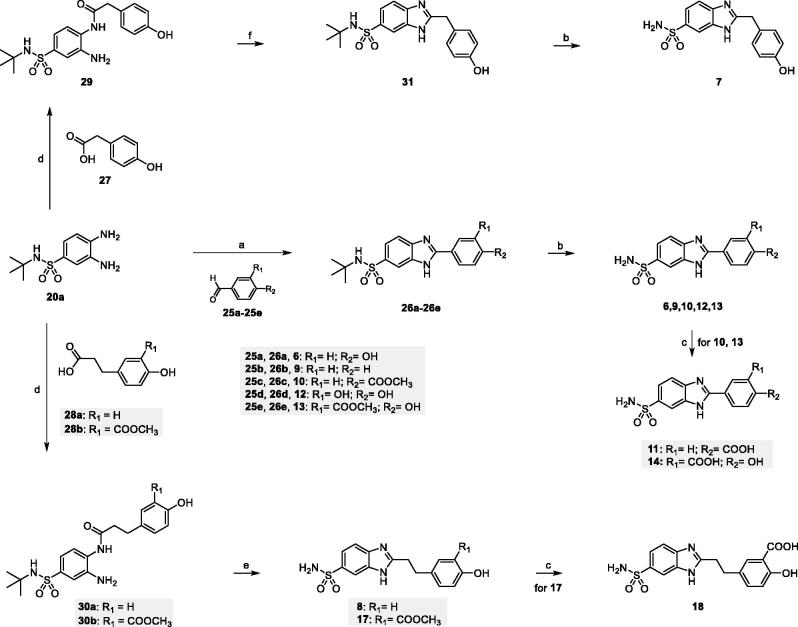
(a) NaHSO_3_, dry DMF, 80 °C, 18 h (65%–83%); (b) DCM/TFA (1:1), r.t., 18–24 h (70–75%); (c) LiOH, THF/H_2_O (1:1) r.t, 3 h (83–93%); (d) EDC hydrochloride, HOBt, NMM, dry DMF, r.t., 18 h (64–90%); (e) *p*-toluenesulfonic acid, toluene, reflux, 6 h (60–65%); (f) MW, AcOH, 80 °C, 30 min (74%).

The synthetic route to 2-aryl-1*H*-benzo[*d*]imidazole-6-sulfonamides **6–14** is outlined in Scheme 2. Condensation of 3,4-diamino-*N*-(*tert*-butyl)benzenesulfonamide **20a** with aldehydes **25a-e** in the presence of NaHSO_3_ in dry DMF at 80 °C gave the 2-aryl-*N*-(tert-butyl)-1*H*-benzo[*d*]imidazole-6-sulfonamides **26a-e** in good yields (65–83%)^38^. Deprotection with trifluoracetic acid at room temperature furnished the primary sulfonamides **6**, **9**, **10**, **12** and **13**. Finally, the carboxylic acid derivatives **11** and **14** were obtained by deprotecting the methyl esters **10** and **13**, respectively, with lithium hydroxide.

Coupling reaction of benzenesulfonamide **20a** with 2-(4-hydroxyphenyl)acetic acid **27** or 3-arylpropanoic acids **28a** and **28b**, in the presence of the peptide coupling reagents hydroxybenzotriazole (HOBt) and 1-ethyl-3–(3-dimethylaminopropyl)carbodiimide hydrochloride (EDC) and *N*-methylmorpholine in dry DMF, furnished the amides **29**, **30a** and **30b** as regioisomeric mixtures, which were purified without separation of regioisomers. Benzimidazoles derivatives **8** and **17** were straightforwardly obtained by *p*-toluenesulfonic acid-mediated cyclization and deprotection of the corresponding 2-amido anilines **30a** and **30b** in refluxing toluene. The carboxylic acid **18** was obtained by deprotecting the methyl ester **17** with lithium hydroxide. Our attempts to cyclize compound **29** using the same reaction conditions were not successful. On the other hand, benzimidazole **31** was obtained in good yield (74%) by using acetic acid under microwave irradiation. Finally, deprotection with trifluoracetic acid at room temperature furnished the primary sulfonamide **7**.

**Scheme 3.**
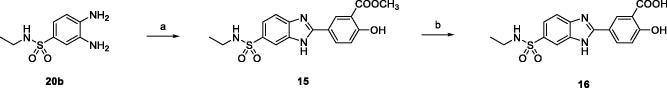
(a) **25e**, NaHSO_3_, dry DMF, 80 °C, 18 h (71%); (b) LiOH, THF/H_2_O (1:1) r.t, 3 h (89%).

*N*-ethyl-2-aryl-1*H*-benzo[*d]*imidazole-6-sulfonamides **15** and **16** were obtained starting from 3,4-diamino-*N*-ethylbenzenesulfonamide **20b** and aldehyde **25e** following the same synthetic strategy used for the preparation of the primary sulfonamide analogs **13** and **14** (Scheme 3).

**Scheme 4.**
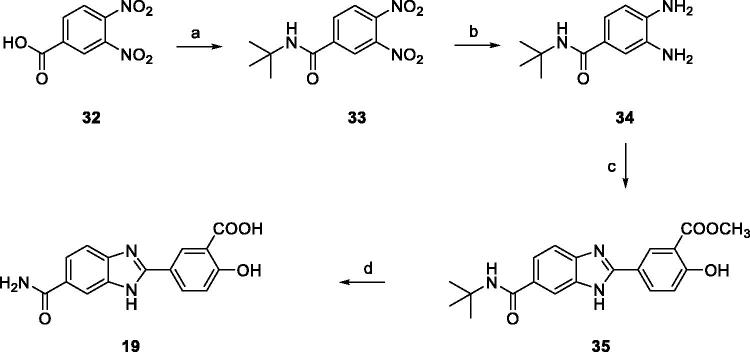
(a) SOCl_2_, reflux, 2 h, then *tert*-butylamine, NEt_3_, THF, 0 °C to r.t., 18 h (84%); (b) ammonium formate, Pd/C 10%, MeOH, reflux, 2 h (94%); (c) **25e**, NaHSO_3_, dry DMF, 80 °C, 18 h (72%); (d) DCM/TFA (1:1), r.t. 24 h, then LiOH, THF/H_2_O (1:1) r.t.,4 h (91%).

Finally, 2–(4-hydroxy-3-carboxy)-phenyl-1*H*-benzo[*d*]imidazole-6-carboxamide **19** was straightforwardly synthesized as described in Scheme 4. After activation of the commercially available 3,4-dinitrobenzoic acid **32** with thionyl chloride, coupling with *tert*-butyl amine yielded 3,4-dinitrobenzamide **33** which was reduced by catalytic hydrogenation with ammonium formate and palladium catalyst to the key intermediate **34**. Condensation with aldehyde **25e** in the presence of NaHSO_3_ gave the benzimidazole derivative **35**. Amide deprotection with trifluoracetic acid and hydrolysis of the methyl ester with LiOH gave the desired compound **19**.

### CA inhibition assays and structure-activity relationship (SAR) considerations

Table 1 lists the enzyme inhibitory activities of the newly synthesized compounds **6–19** against the human (h) CA I, II, IX and XII isoforms, assessed by a stopped-flow CO_2_ hydrase assay [19]. AAZ **1** was used as the standard drug in the assay^18^. Selectivity ratios (SR) for inhibiting the tumor-associated transmembrane isoforms (hCA IX and XII) over the physiologically dominant cytosolic one (hCA II) are also reported for the most active compounds.

**Table 1.** Inhibition data of human CA I, II, IX, and XII isoforms with compounds 6–19 and the standard sulfonamide inhibitor AAZ (1) by a stopped-flow CO_2_ hydrase assay.

Compound		K_i_ (nM)^a^					
		hCA I	hCA II	hCA IX	hCA XII	SR^b^ IX/II	SR^b^ XII/II
**1 (AAZ)**		250	12	25	5.7	0.48	2.1
**6**		237.9	101.2	17.4	44.1	5.8	2.3
**7**		442.1	91.9	73.9	63.8	1.2	1.4
**8**		213.6	47.8	14.4	9.8	3.3	4.9
**9**		92.8	104.1	47.6	78.9	2.2	1.3
**10**		208.2	185.4	29.6	72.2	6.3	2.6
**11**		97.6	133.2	64.3	57.0	2.1	2.3
**12**		169.3	79.0	41.6	8.5	1.9	5.8
**13**		125.5	50.8	2.2	22.3	23.1	2.3
**14**		95.6	68.6	34.2	3.8	2.0	18.1
**15**		>10000	8921.1	390.5	813.1	–	–
**16**		>10000	7582.9	665.1	464.2	–	–
**17**		427.3	52.7	5.9	7.9	8.9	6.7
**18**		441.4	24.8	7.6	4.2	3.3	5.9
**19**		>10000	>10000	>10000	>10000	–	–

a Mean from 3 different assays, by a stopped-flow technique (errors were in the range of ± 5–10% of the reported values).

b SR: Selectivity Ratio.

First, as putative leads for the development of selective CAIs, we synthesized compound **6** and its homologs with a methyl or ethyl linker between the benzimidazole and the phenol moieties (compounds **7** and **8** in Table 1). They all resulted in medium-potency inhibition of the slow cytosolic isoform hCA I, with K_i_ values ranging from 213.6 nM to 442.1 nM. The 4′-hydroxybenzyl derivative **7** showed good inhibition activity for hCA II, IX, and XII but was not selective (K_i_ 91.9 nM for hCA II, 73.9 nM for hCA IX, 63.8 nM for hCA XII). On the other hand, the 4′-hydroxyphenyl (**6**) and 4′-hydroxyphenylethyl (**8**) analogs were effective inhibitors of hCA IX and XII, respectively, with K_i_s in the low nanomolar range (**6**, K_i_ 17.4 nM for hCA IX, 44.1 nM for hCA XII; **8**, K_i_ 14.4 nM for hCA IX, 9.8 nM for hCA XII) basically comparable to those of the reference 1 (K_i_ 25 nM for hCA IX, 5.7 nM for hCA XII), and interesting selectivity ratio (SR) *vs* hCA II.

Based on these data, compound **7** was not further investigated, and a structure-activity relationship (SAR) study was undertaken on **6**. In particular, different substitution patterns on the pendant 2-phenyl ring at 5-position of benzimidazole were investigated (compounds **9–17**). Deletion of the 4′-hydroxy substituent (**9**), as well as its replacement with methoxycarbonyl (**10**) or carboxy (**11**) groups, produces good but unselective inhibitors; in fact, a modest gain (**9**, **11**) or a subsistence (**10**) in activity toward the isoform I and a general decrease in inhibitory potency for hCA II, IX, and XII with respect to the lead **6** were observed, suggesting a precise role played by the hydroxy group in the interaction with the enzyme. This is confirmed by the inhibitory activities showed by compounds **12–14**, where the introduction of an *o*-substituent to the *p*-hydroxy group on the 2-phenyl ring resulted in a moderate increase in activity toward the two cytosolic CA evaluated, and a more considerable improvement for the isoform XII, with compound **14** being the most potent hCA XII inhibitor (K_i_ for hCA XII 3.8 nM), showing also a good SR with respect to hCA II (SR 18.1).

Specifically, the introduction of a second polar group (OH or COOH) at the *ortho* position to the phenol ring positively affected the interaction with isoform XII (compounds **12** and **14**), while the presence of the methoxycarbonyl group (compound **13**) resulted in a minor increase in hCA XII inhibition with respect to parent compound **6**.

Concerning the IX isoform, an exactly opposite trend can be observed for compounds **12–14**. Decoration of the 2-phenyl moiety with a 4′-hydroxy and 3′-methoxycarbonyl groups gives a highly effective hCA IX inhibitor (**13**), which shows low nanomolar K_i_ (2.2 nM), with a relevant gain in activity compared to the reference 1 (K_i_ 25 nM), and good SR vs hCA II. Differently, compounds **12** and **14**, featuring 3′,4′-dihydroxy and 3′-carboxy-4′-hydroxy substituents, although being rather effective hCA IX inhibitors (K_i_ for hCA IX 41.6 nM and 34.2 nM for **12** and **14**, respectively), show a slight decrease in activity with respect to reference compound **6** (K_i_ for hCA IX 17.4 nM).

These data suggested the *ortho*-carboxy phenol ring of compound **14** and the *ortho*-carboxymethyl phenol ring of compound **13** as proper scaffolds for activity and selectivity for hCA XII and hCA IX, respectively. For these derivatives, further SARs were investigated.

First, we explored structural modifications of the 5-sulfonamide moiety, including the insertion of a small alkyl group at the nitrogen atom to produce secondary sulfonamides (compounds **15** and **16**), and replacement of the sulfonamide with a carboxamide (**19**). The obtained compounds proved to be scarcely active or completely inactive inhibitors of all the CA isoforms tested (K_i_s varying from 390.5 nM to micromolar values), strongly supporting the crucial role played by the primary sulfonamide group in the interaction with the enzyme.

Finally, we explored the effect of the combination of the ethyl linker between the benzimidazole and the 2-substituted phenol moieties (compounds **17** and **18** in Table 1). The potency for the isoform hCA I decreased, while hCA II was slightly more (**18**) or equally inhibited (**17**) with respect to related compounds **14** and **13**, respectively. Both **17** and **18** derivatives resulted to be potent hCA IX and hCA XII inhibitors, showing low nanomolar K_i_ values (**17**, K_i_ for hCA IX 5.9 nM, K_i_ for hCA XII 7.9 nM; **18**, K_i_ for hCA IX 7.6 nM, K_i_ for hCA XII 4.2 nM). However, the presence of the ethylene linker between the benzimidazole scaffold and the side phenyl ring abolished the selectivity for hCA IX and XII isoforms, that was observed for compounds **13** and **14**, respectively.

Noteworthy, compounds **17** and **18** are better inhibitors than the phenol derivative **8**, proving the role of the *ortho*-carboxymethyl phenol and the *ortho*-carboxy phenol rings as proper scaffolds in the development of potent and selective hCA IX and hCA XII inhibitors.

### Molecular docking studies

To clarify the reasons for the activities displayed by the newly designed compounds, molecular docking studies were attained. Docking calculations were performed using a protocol already successfully applied in our previous work on CA inhibitors^39^. Namely, AutoDock4.2 (AD4)^30^^,^^37^ was employed together with the AutoDock4(Zn) forcefield^31^, which was specifically designed to accurately predict the binding interactions of ligands docking to zinc metalloproteins.

**Figure 2.**
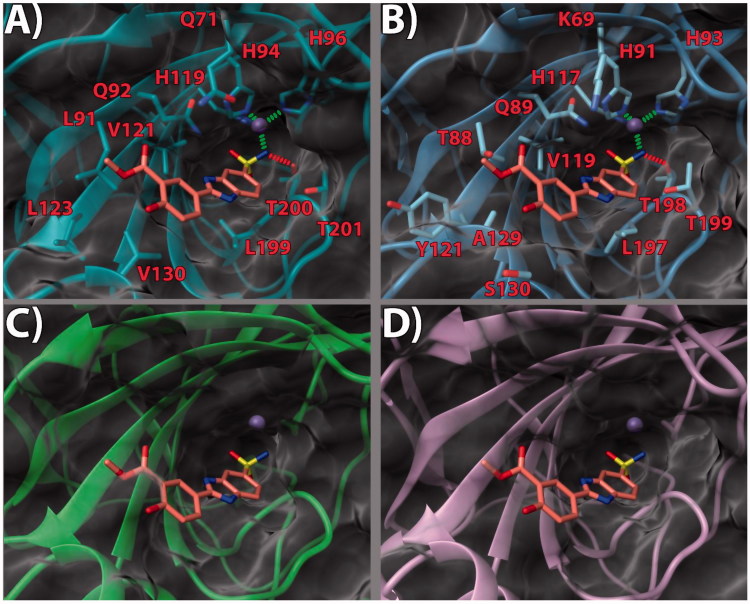
(a) **13**/hCA IX (PDB 5FL4) theoretical complex as calculated by docking simulations. The protein is shown as cyan ribbons and sticks while the ligand as salmon sticks. Critical residues are labeled. H-bonds are depicted as red dashed lines while coordination bonds as green dashed lines. (b) **13** hCA IX theoretical binding pose within the hCA XII (PDB 5MSA) X-ray structure^29^. The protein is shown as light blue ribbons and sticks while the ligand as salmon sticks. Critical residues are labeled. H-bonds are depicted as red dashed lines while coordination bonds as green dashed lines. (c) **13** hCA IX theoretical binding pose within the hCA I (PDB 6F3B) structure. The protein is shown as green ribbons and its molecular surface in transparent gray. The ligand is represented as salmon sticks. (d) **13** hCA IX theoretical binding pose within the hCA II (PDB 3K34) structure. The protein is shown as pink ribbons and its molecular surface in transparent gray. The ligand is represented as salmon sticks. The images were rendered using the UCSF Chimera software^30^.

**Figure 3.**
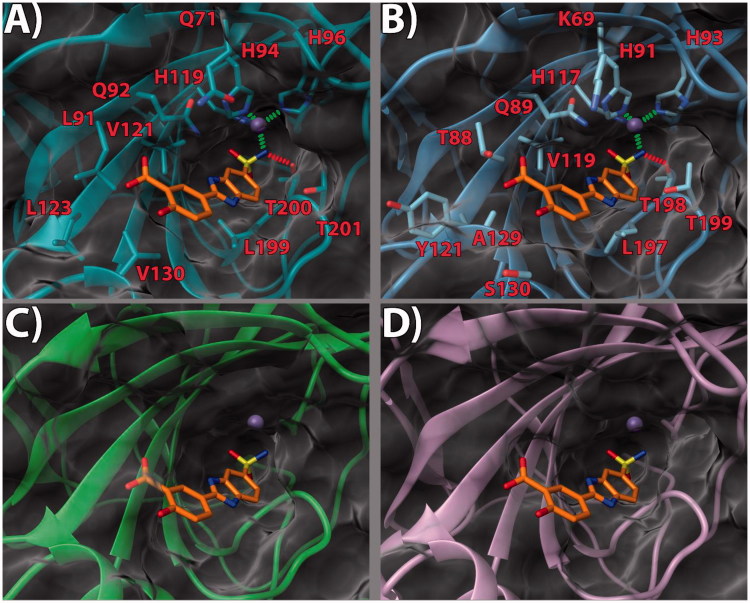
(a) **14**/hCA IX (PDB 5FL4) theoretical complex as calculated by docking simulations. The protein is shown as cyan ribbons and sticks while the ligand as orange sticks. Critical residues are labeled. H-bonds are depicted as red dashed lines while coordination bonds as green dashed lines. (b) **14** hCA IX theoretical binding pose within the hCA XII (PDB 5MSA) X-ray structure. The protein is shown as light blue ribbons and sticks while the ligand as orange sticks. Critical residues are labeled. H-bonds are depicted as red dashed lines while coordination bonds as green dashed lines. (c) **14** hCA IX theoretical binding pose within the hCA I (PDB 6F3B) X-ray structure. The protein is shown as green ribbons and its molecular surface as transparent gray. The ligand is shown as orange sticks. (d) **14** hCA IX docked binding pose within the hCA II (PDB 3K34) structure. The protein is shown as pink ribbons and its molecular surface in transparent gray. The ligand is depicted as orange sticks. The images were rendered using the UCSF Chimera software^30^.

**Figure 4.**
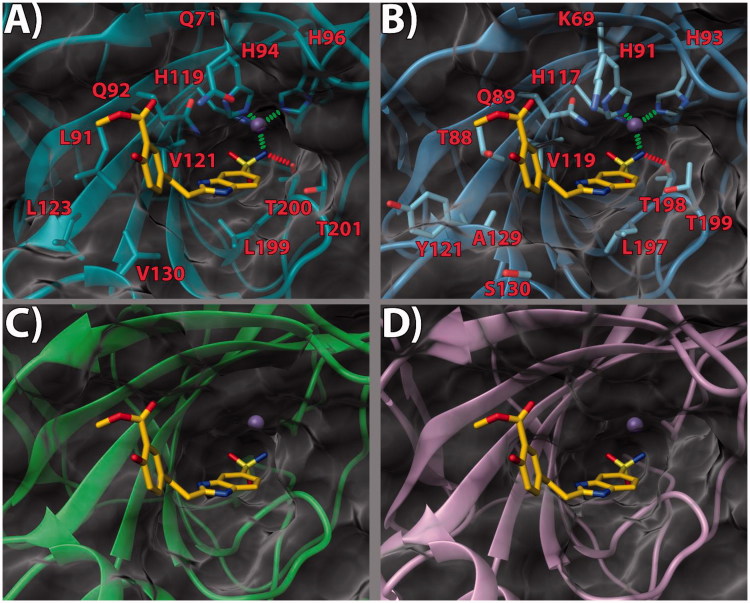
(a) **17**/hCA IX (PDB 5FL4) theoretical complex as calculated by docking simulations. The protein is shown as cyan ribbons and sticks while the ligand as yellow sticks. Critical residues are labeled. H-bonds are depicted as red dashed lines while coordination bonds as green dashed lines. (b) **17** hCA IX theoretical binding pose within the hCA XII (PDB 5MSA) X-ray structure. The protein is shown as light blue ribbons and sticks while the ligand as yellow sticks. Critical residues are labeled. H-bonds are depicted as red dashed lines while coordination bonds as green dashed lines. (c) **17** hCA IX theoretical binding pose within the hCA I (PDB 6F3B) X-ray structure. The protein is shown as green ribbons and its molecular surface as transparent gray. The ligand is shown as yellow sticks. (d) **17** hCA IX docked binding pose within the hCA II (PDB 3K34) structure. The protein is shown as pink ribbons and its molecular surface in transparent gray. The ligand is depicted as yellow sticks. The images were rendered using the UCSF Chimera software^30^.

Ligands **13**, **14** and **17** were selected for the in silico experiments as representative of the whole set. These compounds were first docked in the active site of hCA IX. For the latter, a high-resolution X-ray crystal structure bound to a small molecule inhibitor (PDB code 5FL4)^32^ was chosen. According to our theoretical model (Figures 2(A), 3(A) and 4(A)), in the three inspected ligands, the negative nitrogen of the sulfonamide group chelates the zinc ion of the active site. The sulfonamide also engages an H-bond with the backbone of T200. Furthermore, the benzimidazole nitrogen is in a potential H-bond accepting position with Q92 side chain. The benzimidazole core also engages in contacts with the V121, L199, and T201 sidechains (Figure 2(A), Figure 3(A) and Figure 4(A)). Notably, it would appear that the pendant 2-phenyl ring, in the three ligands, points towards what has been defined as a “selectivity hot spot” in CAs^40^ (Figures 2(A), 3(A) and 4(A)): a high-variability region in CAs binding site, that can be exploited for the rational design of selective compounds among different CAs. Here, ligand **13** phenyl ring and its 3′-methoxycarbonyl moiety are able to establish favorable contacts with the lipophilic sidechains of L91 and V130 (Figure 2(A)). On the other hand, the 4′-hydroxy group is pointing outside of the binding site, probably establishing a network of stabilizing H-bonding interactions with the solvent water molecules (Figure 2(A)). It can be argued that this accounts for the higher potency displayed by compounds featuring the 4′-hydroxy group. The same holds true for compound **14**. As for ligand **17**, while the benzimidazole core binding mode is conserved, the ethyl linker engenders greater flexibility which allows the side phenyl ring to expand further into the hotspot gorge. This allows enhancing the positive contacts with L91 and V130. Moreover, an additional H-bond between the 3′-methoxycarbonyl group and Q71 is formed (Figure 4(A)).

With the aim of rationalizing the selectivity of the compounds, the crystal structures of hCA I (PDB 6F3B)^34^, hCA II (PDB 3K34)^35^, and hCA XII (PDB 5MSA)^36^ were downloaded and their binding sites analyzed. To ascertain how the predicted docked poses of ligands **13**, **14** and **17** in the hCA IX active site would fit in the other CAs, the 3 D structures of the enzymes were superimposed. This analysis revealed that the recognition pattern achieved for hCA IX, conducive of potent enzyme inhibition, is unlikely to be confirmed for hCA I and hCA II due to major steric clashes (Figures 2(C,D), 3(C,D), and 4(C,D)). Indeed, their binding sites in the hot spot region feature bulky substituents that would unfavorably affect the binding mode of the compounds, especially in the case of ligand **17 (**Figure 4(C,D)).

Conversely, hCA XII and hCA IX binding sites share a higher degree of homology. As such, the binding poses found for **13**, **14** and **17** in hCA IX also fit in hCA XII (Figures 2(B) and 3(B)). Still, few key differences in the hot spot region can be found. Specifically, some hydrophobic residues in hCA IX are replaced by polar ones (L91, L123, V130 in CA IX become T88, Y121, S130 in hCA XII, respectively). Importantly, in hCA XII the positive K69 takes the place of Q71 in hCA IX. It is possible to infer that the more hydrophilic and positively charged binding site of hCA XII provides a better fit for compounds with a 3′-carboxyl group on the pendant 2-phenyl ring (see compound **14**, Figure 3(B)). Instead, compounds bearing the 3′-methoxycarbonyl moiety can interact more favorably with the lipophilic and neutral hot spot region of hCA IX. Purportedly, the presence of the ethyl linker grants the possibility for the ligand to maximize the favorable interactions in hCA IX and hCA XII, with both substitution patterns on the 2-phenyl ring, the 3′-carboxyl (**18**) or the 3′-methoxycarbonyl (**17**) moiety. On the other hand, the same ethyl linker, by enhancing the ligand flexibility, should also allow for a better fit into the hCA I and hCA II isoform structures, thereby negatively impacting on the ligand selectivity profile (Figures 2, 3 and 4).

## Conclusions

Several Schiff bases and secondary amines incorporating aromatic sulfonamide moieties in their structure has been extensively studied as CAIs. Starting from these classes of compounds and according to the frozen analog approach, we designed a series of derivatives featuring the 2-substituted-benzimidazole-6-sulfonamide scaffold, a chemical template only scarcely exploited in the CAIs’ medicinal chemistry field. A library of 14 derivatives was synthesized and tested for their enzyme inhibitory activity against the physiologically relevant human CA I, II, IX, and XII isoforms. Computational studies were attained to rationalize the SAR in terms of inhibitory activity and selectivity profile.

Of note, the identification of a number of newly synthesized derivatives featuring high potency against the hCA IX and or XII isoforms, combined with promising selectivity profiles. These findings could result interesting for the development of novel anticancer agents with limited side effects. Indeed, hCA IX and XII enzymes have recently emerged as excellent targets for the design of novel therapeutic strategies for cancer, due to their involvement in the tumor cells survival as well as in insurgence of resistance to classical anticancer protocols. Extensive SAR analysis and cellular studies are ongoing to increase the knowledge within this series of CAIs inhibitors.

## Supplementary Material

Supplemental Material
