# Supplementary material for: Novel 2-substituted-benzimidazole-6-sulfonamides as carbonic anhydrase inhibitors: synthesis, biological evaluation against isoforms I, II, IX and XII and molecular docking studies
Source: J Enzyme Inhib Med Chem. 2019 Sep 20;34(1):1697–710. doi: 10.1080/14756366.2019.1666836 (PMC6758606; doi:10.1080/14756366.2019.1666836)
Supplement: Supplemental Material [file IENZ_A_1666836_SM9509.pdf]

Supplementary Data

Novel 2-substituted-benzimidazole-6-sulfonamides as carbonic anhydrase inhibitors: synthesis, biological evaluation against isoforms I, II, IX and XII and molecular docking studies

Ciro Milite, [1,a] Giorgio Amendola, [2,a] Alessio Nocentini, [3] Silvia Bua, [3] Alessandra Cipriano, [1,4] Elisabetta Barresi, [5] Alessandra Feoli, [1] Ettore Novellino, [6] Federico Da Settimo, [5] Claudiu T. Supuran, [3] Sabrina Castellano, [1, *] Sandro Cosconati, [2,**] and Sabrina Taliani [5]

[1] *Department of Pharmacy, Epigenetic Med Chem Lab, University of Salerno, via Giovanni Paolo II 132, I-84084 Fisciano (SA), Italy*

[2] *DiSTABiF, Università della Campania Luigi Vanvitelli, Via Vivaldi 43, 81100 Caserta, Italy.*

[3] *NEUROFARBA Department, Sezione di Scienze Farmaceutiche e Nutraceutiche, Università degli Studi di Firenze, Via Ugo Schiff 6, 50019 Sesto Fiorentino (Florence), Italy;*

[4] *PhD Program in Drug Discovery and Development, University of Salerno, Via Giovanni Paolo II 132, I-84084 Fisciano (SA), Italy*

[5] *Department of Pharmacy, Universy of Pisa, Via Bonanno 6, 56126 Pisa, Italy.*

[6] *Department of Pharmacy, University Federico II of Naples, Via D. Montesano 49, 80131 Naples, Italy*

Table of Contents:

- Preparation of compound **28b**: **S3**
- ^{1}H-NMR and ^{13}C-NMR spectra of compound **6–19**: **S5–S32**

Preparation of *3-(4-Hydroxy-3-(methoxycarbonyl)phenyl)propanoic acid* (28b)

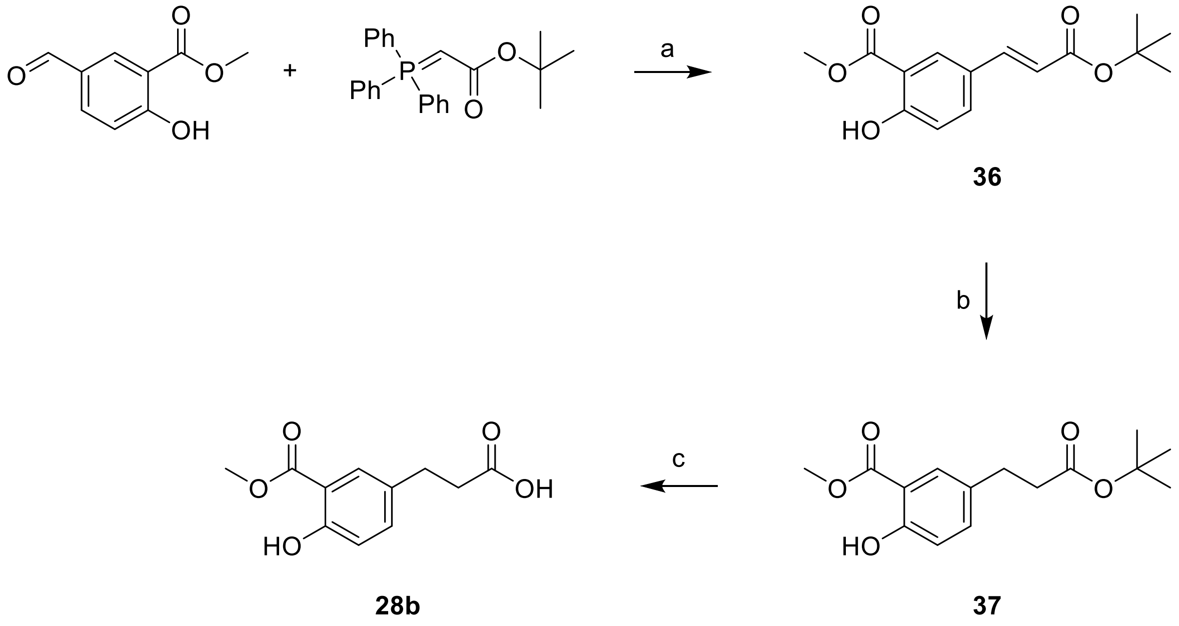

(a) Toluene, 18 h (98%); (b) ammonium formate, Pd/C 10 %, MeOH, reflux, 4 h (98%); (c) DCM/TFA (9:1), r.t. 3 h (99%).

*Methyl (E)-5-(3-(tert-butoxy)-3-oxoprop-1-en-1-yl)-2-hydroxybenzoate (**36**)*

To a suspension of methyl 5-formyl-2-hydroxybenzoate (400 mg, 2.22 mmol) in 25 mL of toluene, tert-butyl(triphenylphosphoranylidene)acetate (1.67 g, 4.44 mmol) was added and the resulting mixture was stirred at room temperature for 18h. Solvent was evaporated and the crude material was purified by silica gel chromatography (DCM/EtOAc, 9/1) yielding **36** as a white solid (605 mg, 98%). ^{1}H NMR (400 MHz, $CDCl_3$) δ 10.95 (s, 1H), 8.00 (d, J = 2.3 Hz, 1H), 7.63 (dd, J = 8.7, 2.3 Hz, 1H), 7.51 (d, J = 15.9 Hz, 1H), 6.99 (d, J = 8.7 Hz, 1H), 6.27 (d, J = 16.0 Hz, 1H), 3.98 (s, 3H), 1.53 (s, 9H). ESI m/z: 279 $[M+H]^+$.

*Methyl 5-(3-(tert-butoxy)-3-oxopropyl)-2-hydroxybenzoate (**37**)*

To a stirred suspension of **36** (550 mg, 1.97 mmol) in 120 mL of MeOH, ammonium formate (1.24 g, 19.7 mmol) and palladium on carbon 10 % wt. (36 mg) were added. The resulting mixture was heated at reflux for 4 h. After cooling, the mixture was filtered, and the solvent evaporated under reduced pressure. The crude material was taken up with 100 mL of water and extracted with EtOAc (3 x 60 mL). The combined organic phases were washed with brine, dried over Na_2SO_4, filtered and evaporated. The product, obtained as a white solid (540 mg, 98%), was used for the next step without further purification. ^{1}H NMR (400 MHz, $CDCl_3$) δ 10.62 (s, 1H), 7.69 (d, 1H, J = 1.5 Hz), 7.33 (d, J = 8.5 Hz, 2H), 6.93 (dd, J = 8.5, 1.5 Hz, 1H), 3.96 (s, 3H), 2.87 (t, J = 7.5 Hz, 2H), 2.53 (t, J = 7.6 Hz, 2H), 1.44 (s, 9H). ESI m/z: 281 $[M+H]^+$.

*3-(4-Hydroxy-3-(methoxycarbonyl)phenyl)propanoic acid (**28b**)*

Compound **37** (500 mg, 1.78 mmol) was dissolved in a mixture of DCM/TFA (5 mL, 9/1) and the resulting solution was stirred at room temperature for 3 h. Solvent was evaporated yielding essentially pure **28b** (400 mg, 99%) as a white solid. ^{1}H NMR (400 MHz, $CDCl_3$) δ 10.65 (s, 1H), 7.71 (s, 1H), 7.34 (d, J = 8.5 Hz, 1H), 6.95 (d, J = 8.5 Hz, 1H), 3.97 (s, 3H), 2.92 (t, J = 7.6 Hz, 2H), 2.68 (t, J = 7.6 Hz, 2H). ESI m/z: 225 $[M+H]^+$.

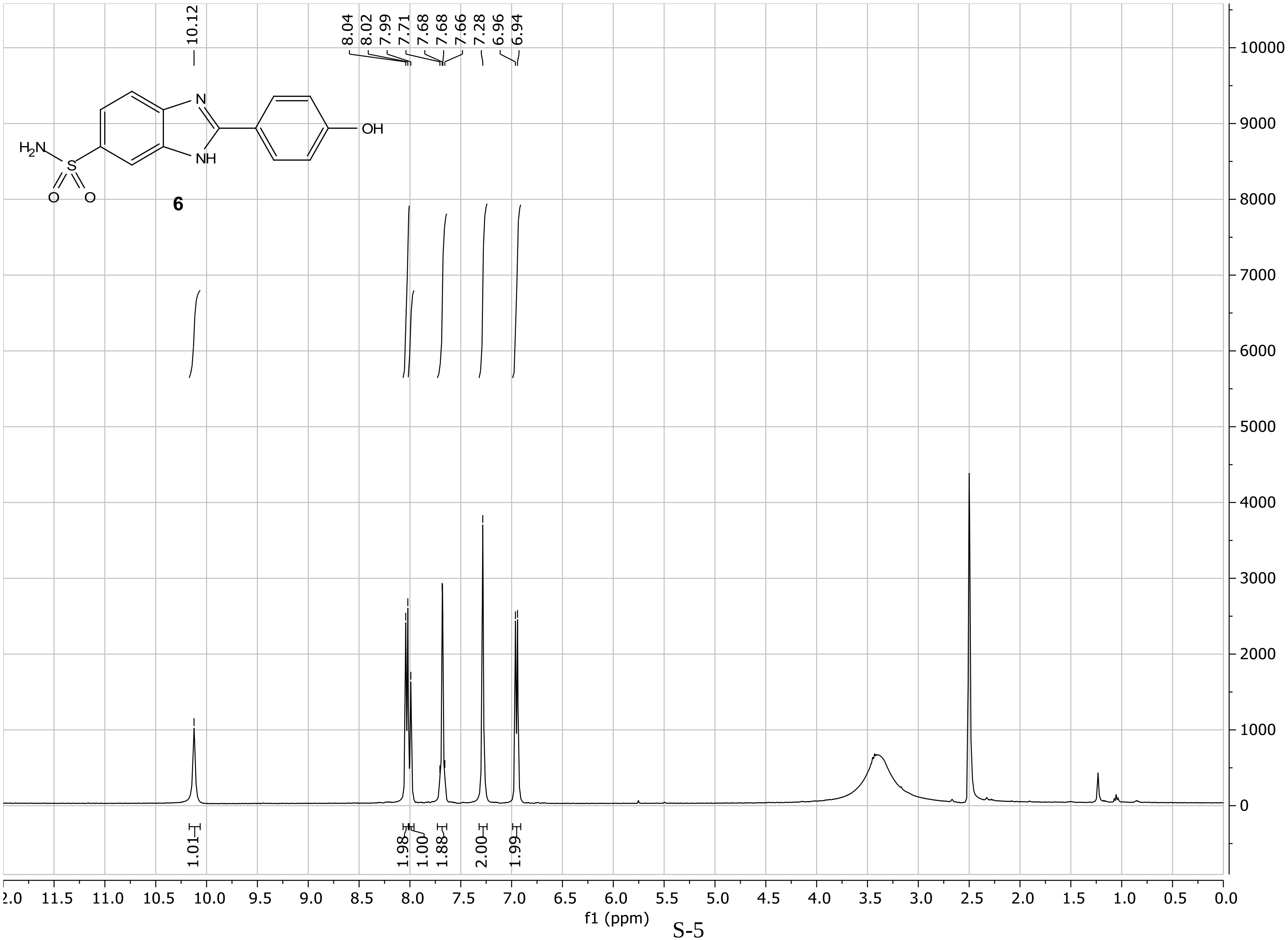
10.12
8.04
8.02
7.99
7.71
7.68
7.68
7.66
7.28
6.96
6.94
H2N
S
O
O
N
NH
OH
6
1.01
1.98
1.00
1.88
2.00
1.99
f1 (ppm)

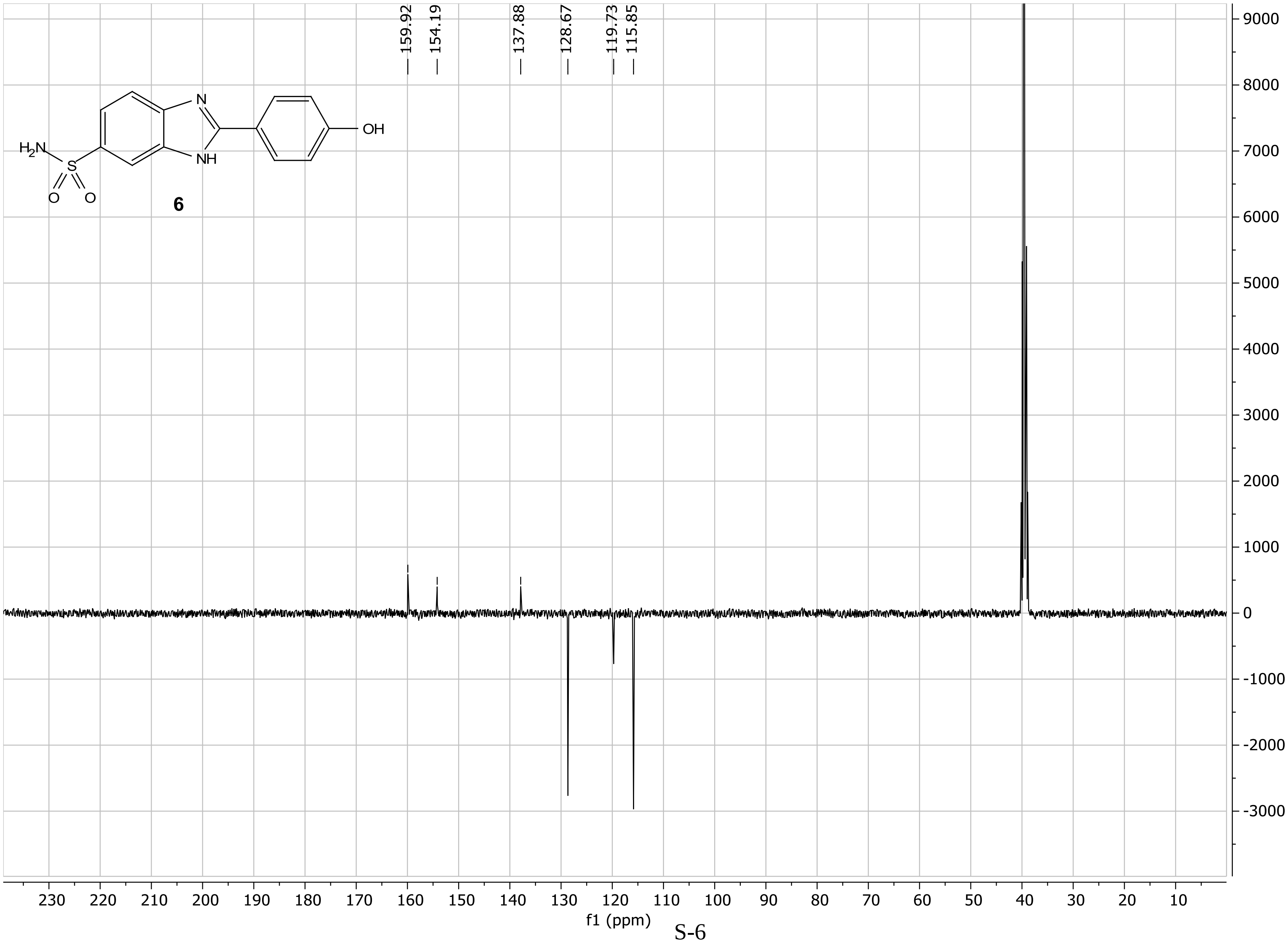
159.92
154.19
137.88
128.67
119.73
115.85
H2N
S
O
O
N
NH
OH
6
f1 (ppm)

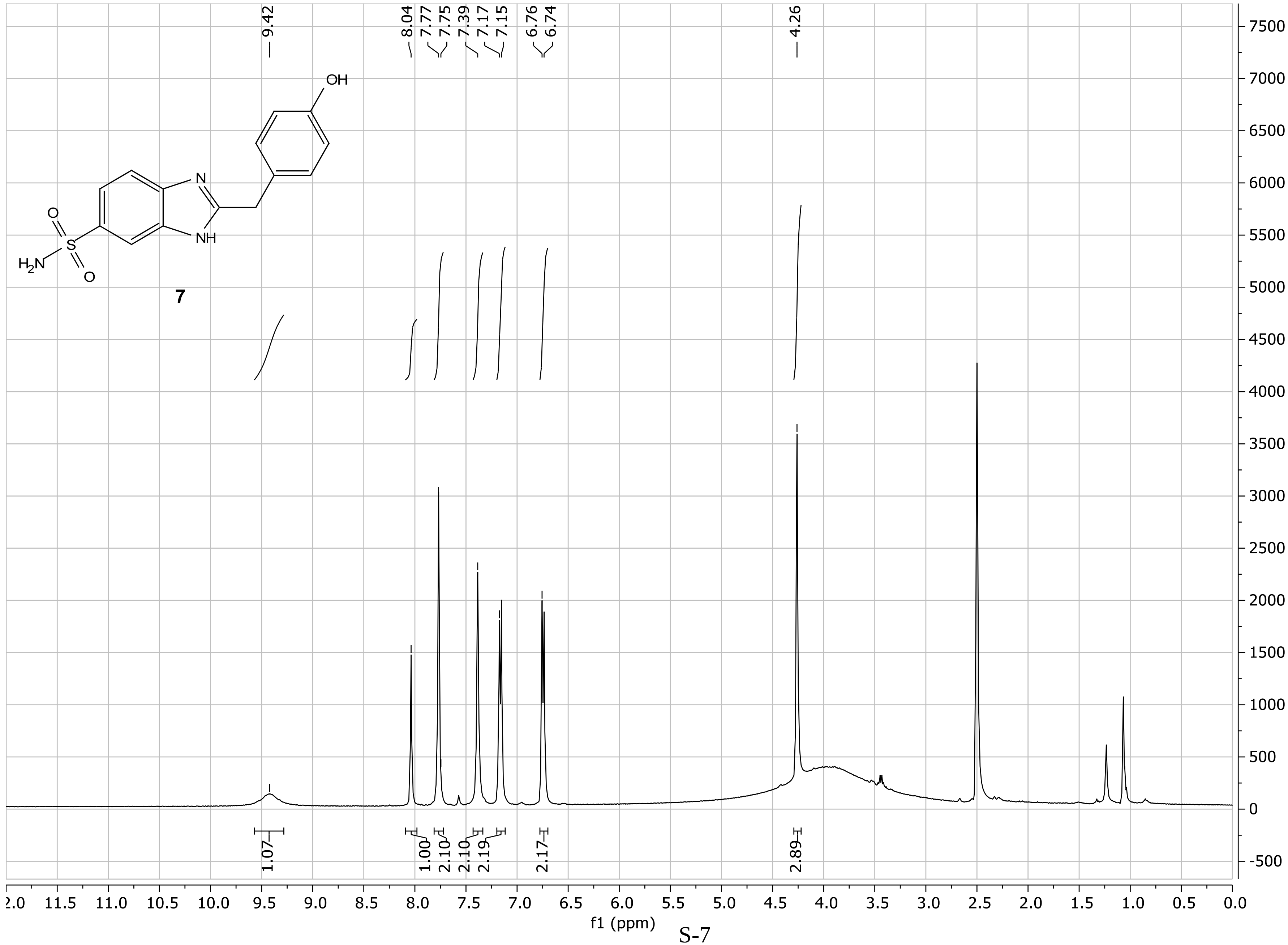
9.42
8.04
7.77
7.75
7.39
7.17
7.15
6.76
6.74
4.26
OH
N
NH
O
S
H2N
O
7
1.07
1.00
2.10
2.10
2.19
2.17
2.89
7500
7000
6500
6000
5500
5000
4500
4000
3500
3000
2500
2000
1500
1000
500
0
-500
11.5 11.0 10.5 10.0 9.5 9.0 8.5 8.0 7.5 7.0 6.5 6.0 5.5 5.0 4.5 4.0 3.5 3.0 2.5 2.0 1.5 1.0 0.5 0.0
f1 (ppm)

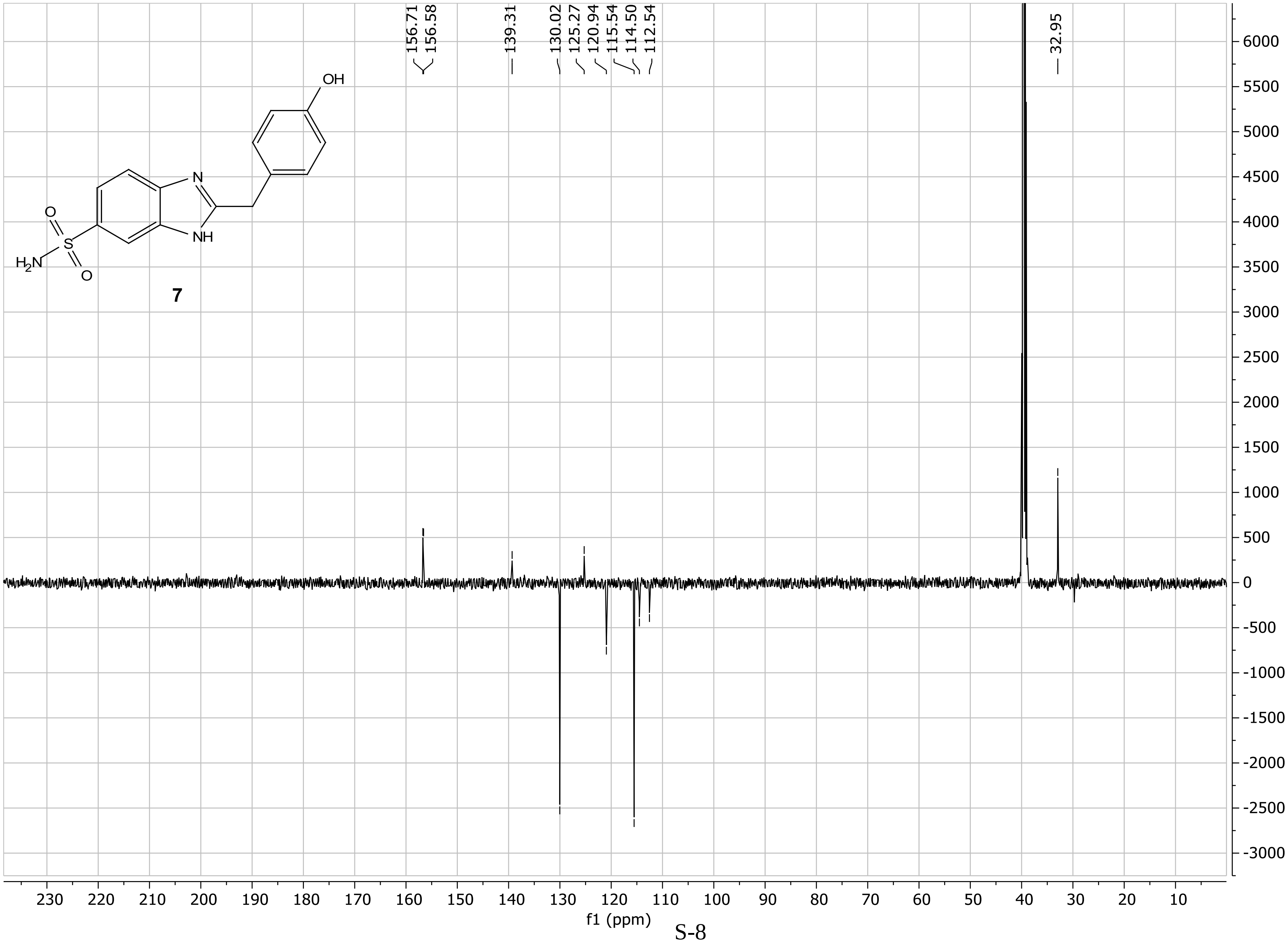
156.71
156.58
139.31
130.02
125.27
120.94
115.54
114.50
112.54
32.95
OH
N
NH
O
S
H_2N
O
7
f1 (ppm)

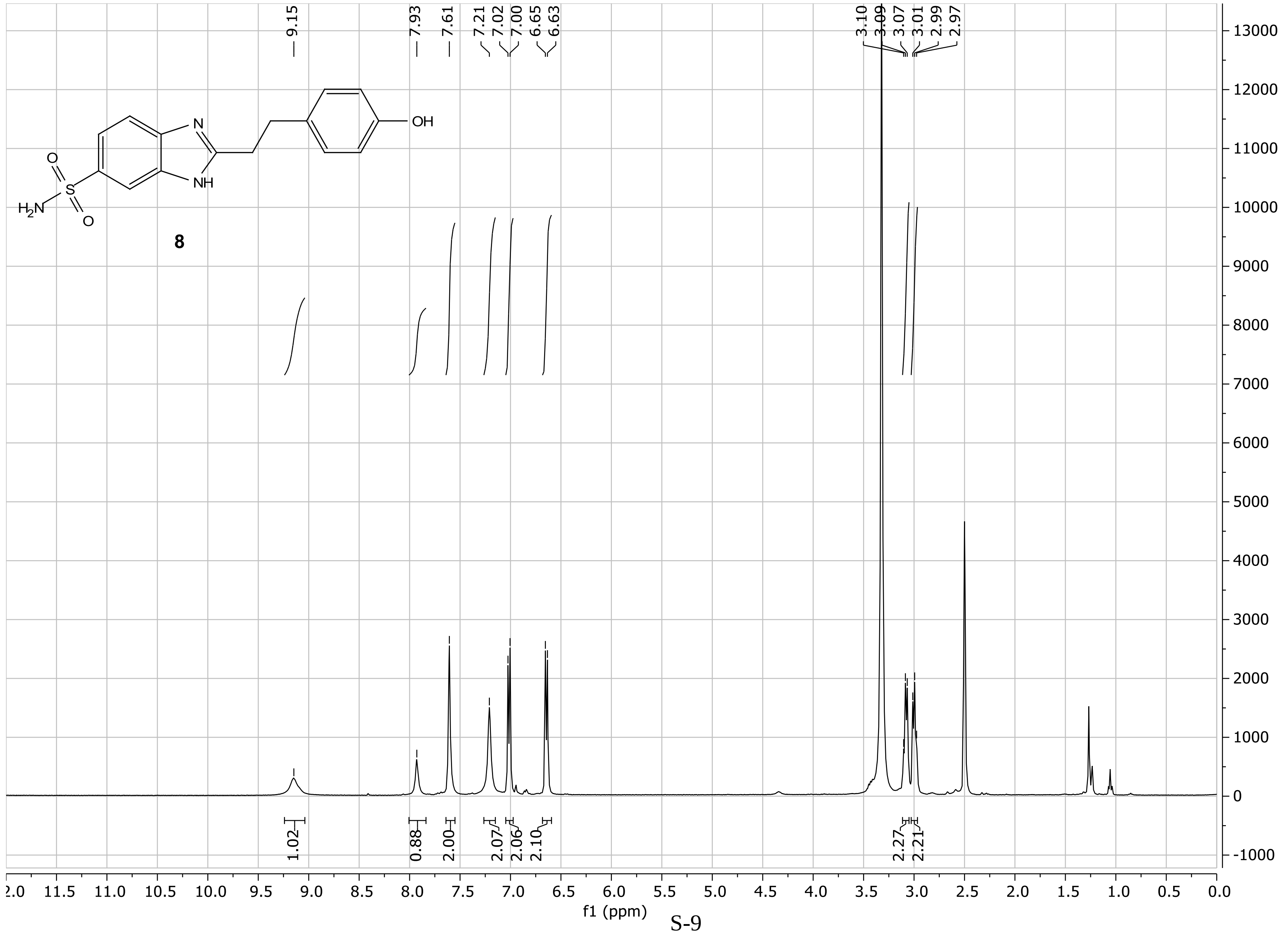

9.15
7.93
7.61
7.21
7.02
7.00
6.65
6.63
3.10
3.09
3.07
3.01
2.99
2.97
OH
N
NH
O
S
H2N
O
8
1.02
0.88
2.00
2.07
2.06
2.10
2.27
2.21
f1 (ppm)

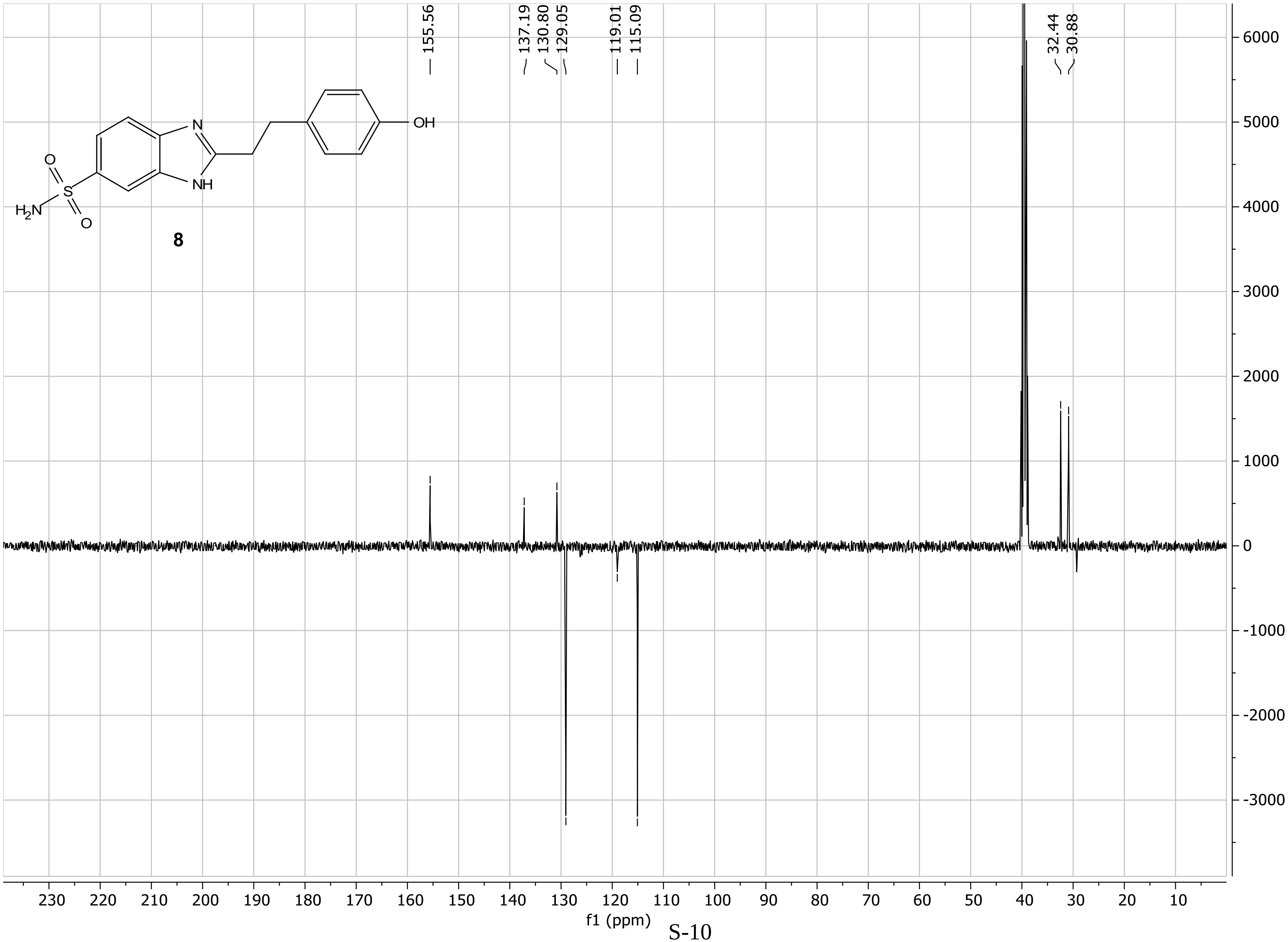
155.56
137.19
130.80
129.05
119.01
115.09
32.44
30.88
OH
N
NH
O
S
O
H2N
8
6000
5000
4000
3000
2000
1000
0
-1000
-2000
-3000
230
220
210
200
190
180
170
160
150
140
130
120
110
100
90
80
70
60
50
40
30
20
10
f1 (ppm)

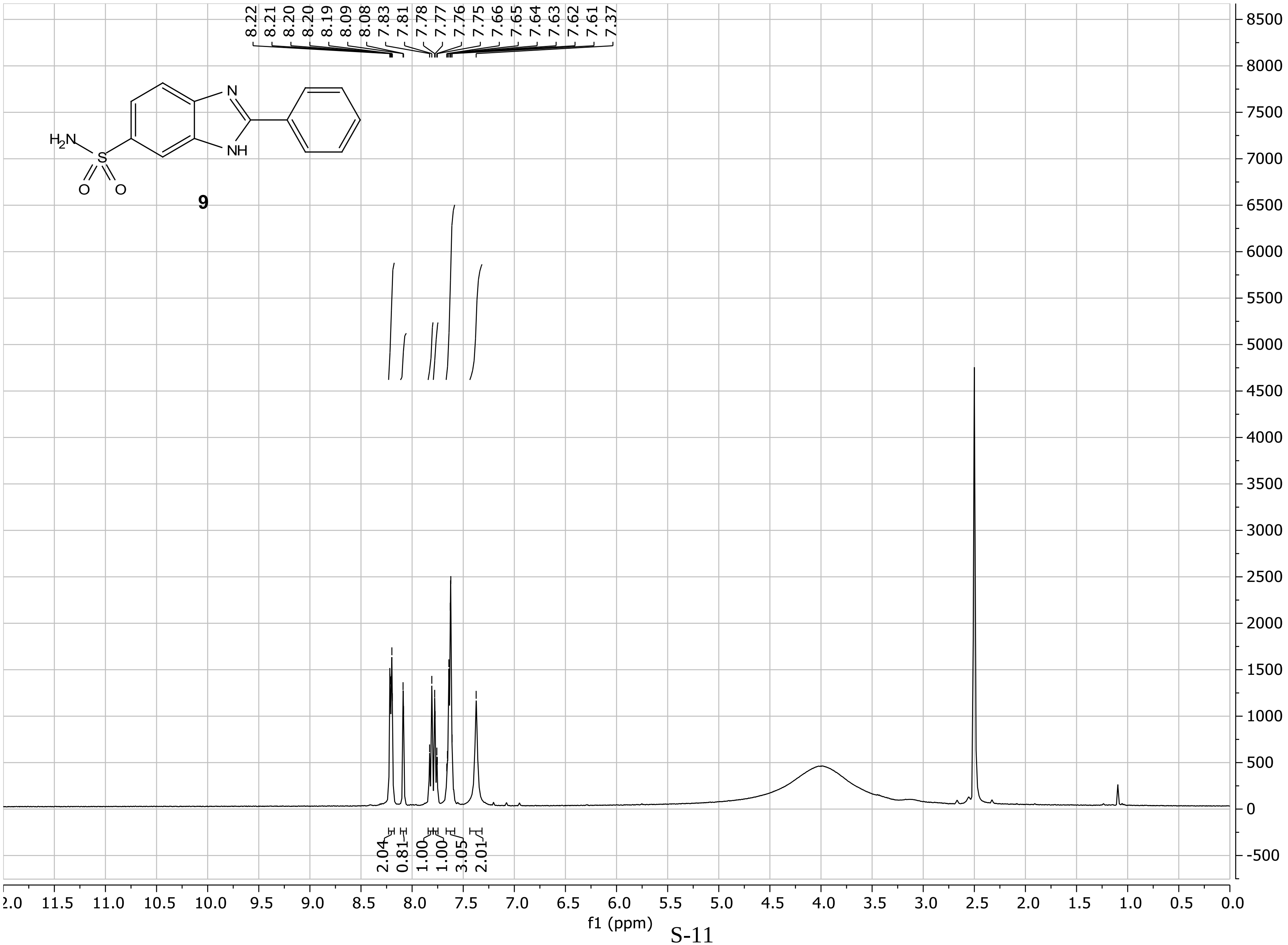
8.22
8.21
8.20
8.20
8.19
8.09
8.08
7.83
7.81
7.78
7.77
7.76
7.75
7.66
7.65
7.64
7.63
7.62
7.61
7.37
9
2.04
0.81
1.00
1.00
3.05
2.01
f1 (ppm)

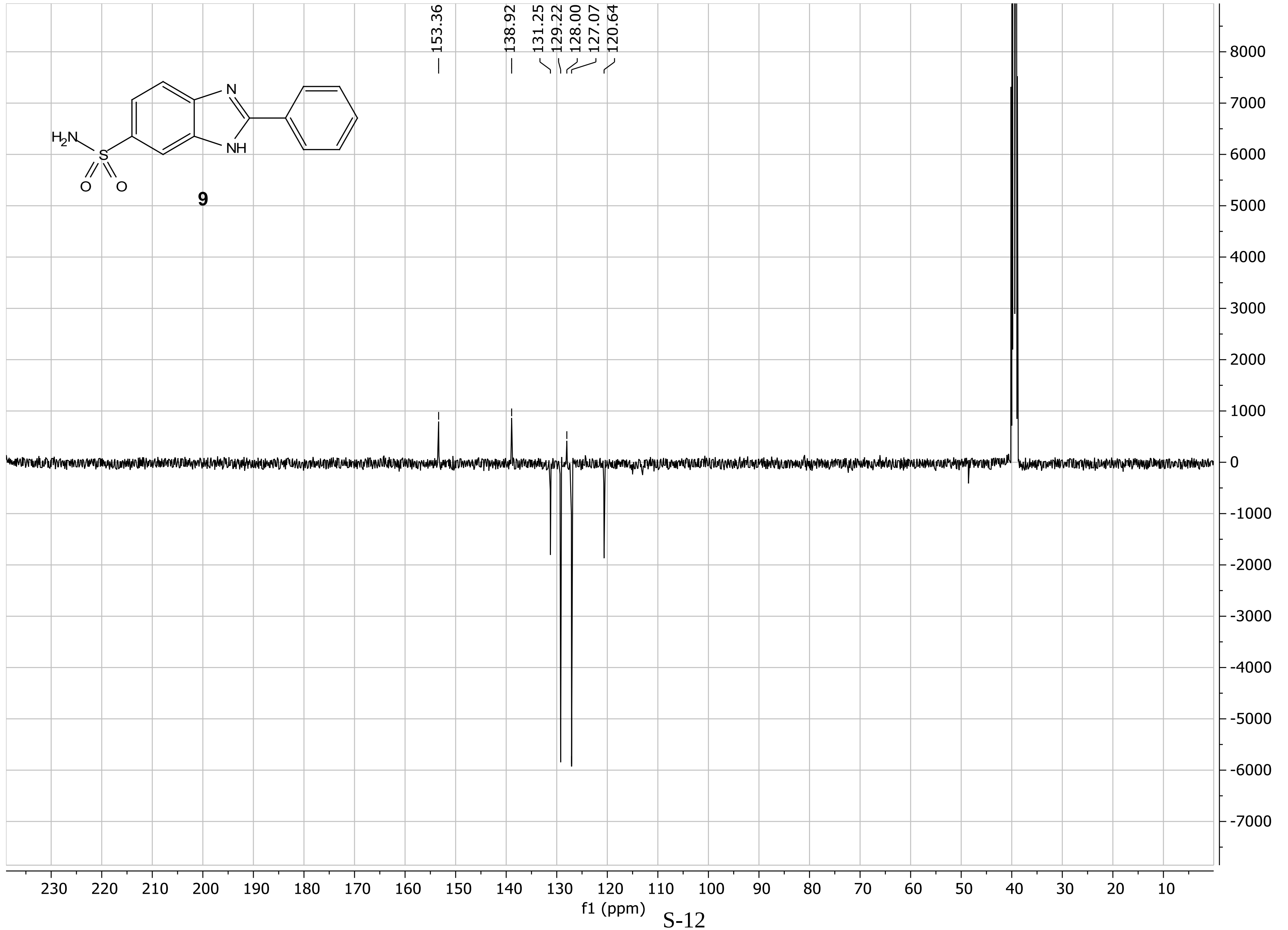
153.36
138.92
131.25
129.22
128.00
127.07
120.64
H2N
S
O
O
N
NH
9
8000
7000
6000
5000
4000
3000
2000
1000
0
-1000
-2000
-3000
-4000
-5000
-6000
-7000
230 220 210 200 190 180 170 160 150 140 130 120 110 100 90 80 70 60 50 40 30 20 10
f1 (ppm)

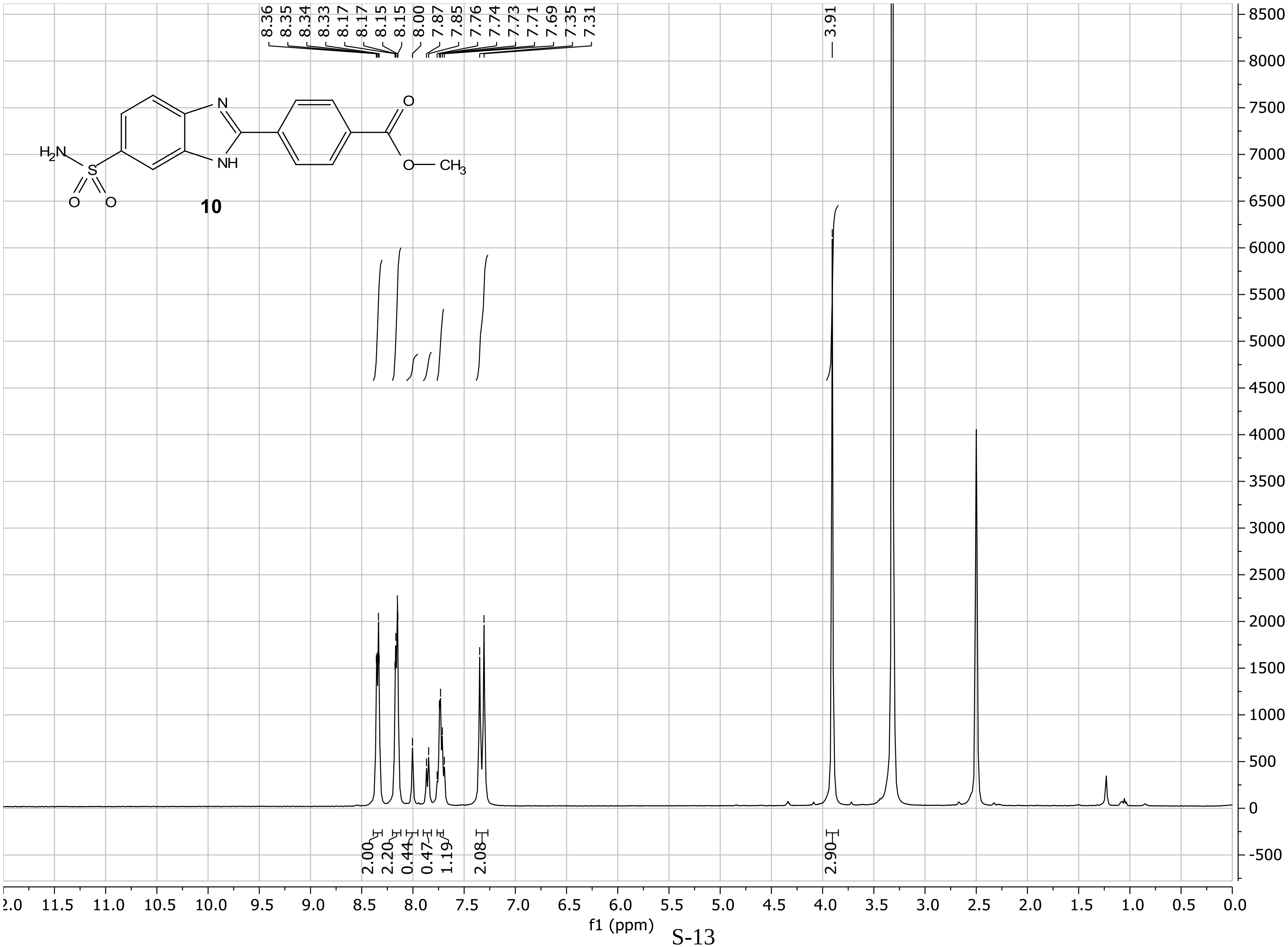

8.36
8.35
8.34
8.33
8.17
8.17
8.15
8.15
8.00
7.87
7.85
7.76
7.74
7.73
7.71
7.69
7.35
7.31
3.91
H2N
S
O
O
N
NH
O
O
CH3
10
2.00
2.20
0.44
0.47
1.19
2.08
2.90
f1 (ppm)

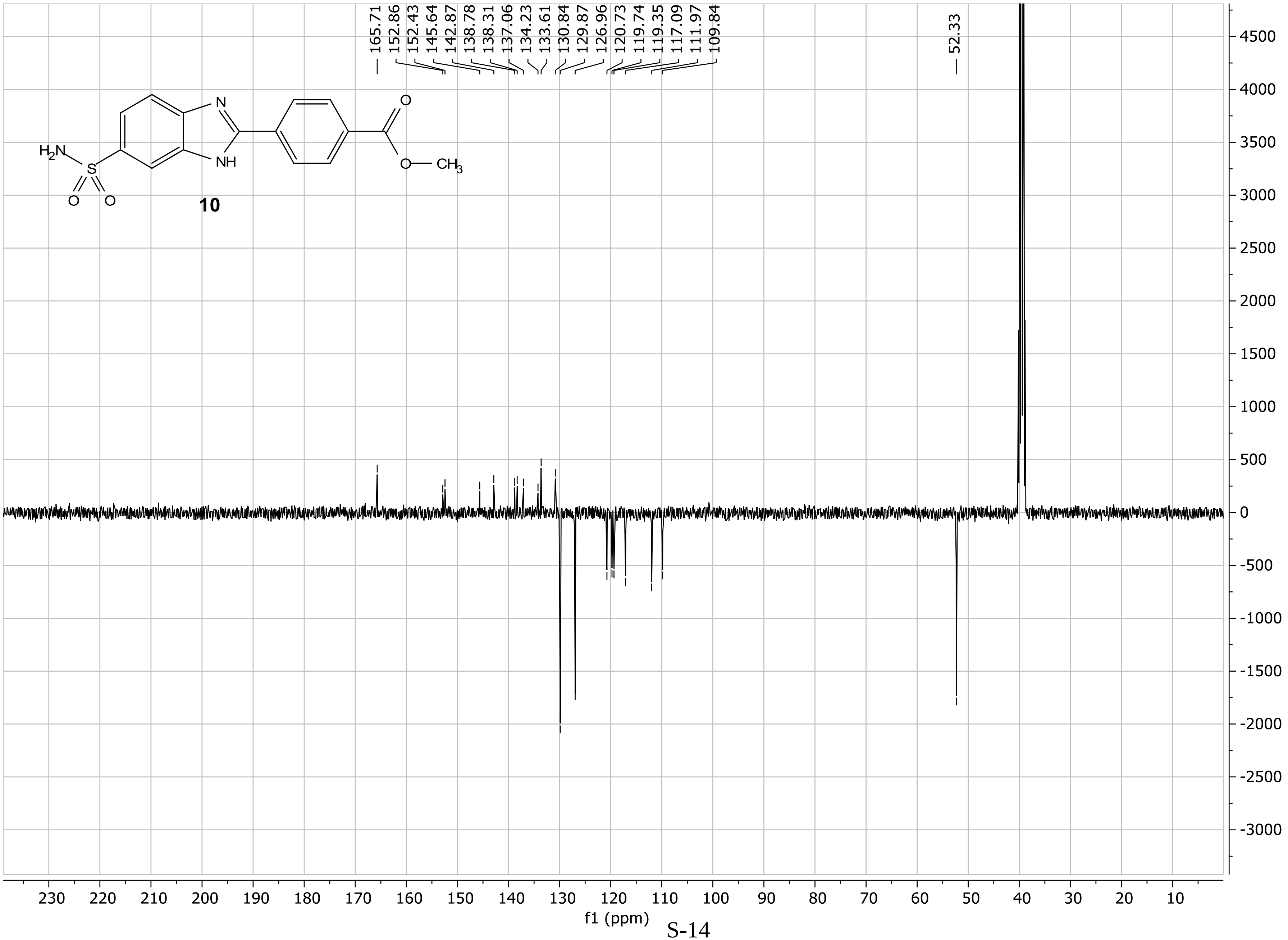
165.71
152.86
152.43
145.64
142.87
138.78
138.31
137.06
134.23
133.61
130.84
129.87
126.96
120.73
119.74
119.35
117.09
111.97
109.84
52.33
H_2N
S
O
O
N
NH
O
O
CH_3
10
4500
4000
3500
3000
2500
2000
1500
1000
500
0
-500
-1000
-1500
-2000
-2500
-3000
230 220 210 200 190 180 170 160 150 140 130 120 110 100 90 80 70 60 50 40 30 20 10
f1 (ppm)

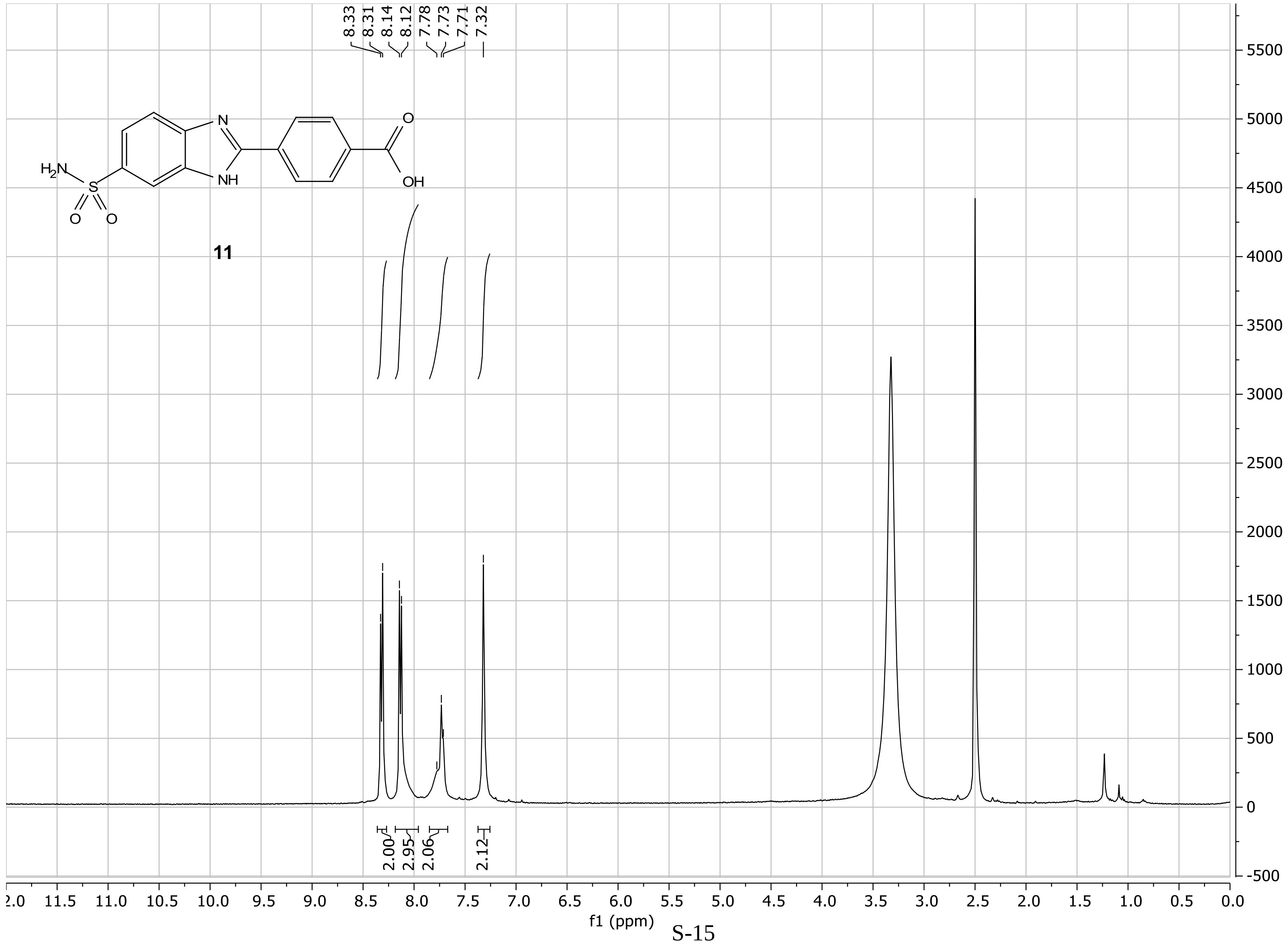
8.33
8.31
8.14
8.12
7.78
7.73
7.71
7.32
H2N
S
O
O
N
NH
O
OH
11
2.00
2.95
2.06
2.12
5500
5000
4500
4000
3500
3000
2500
2000
1500
1000
500
0
-500
11.5 11.0 10.5 10.0 9.5 9.0 8.5 8.0 7.5 7.0 6.5 6.0 5.5 5.0 4.5 4.0 3.5 3.0 2.5 2.0 1.5 1.0 0.5 0.0
f1 (ppm)

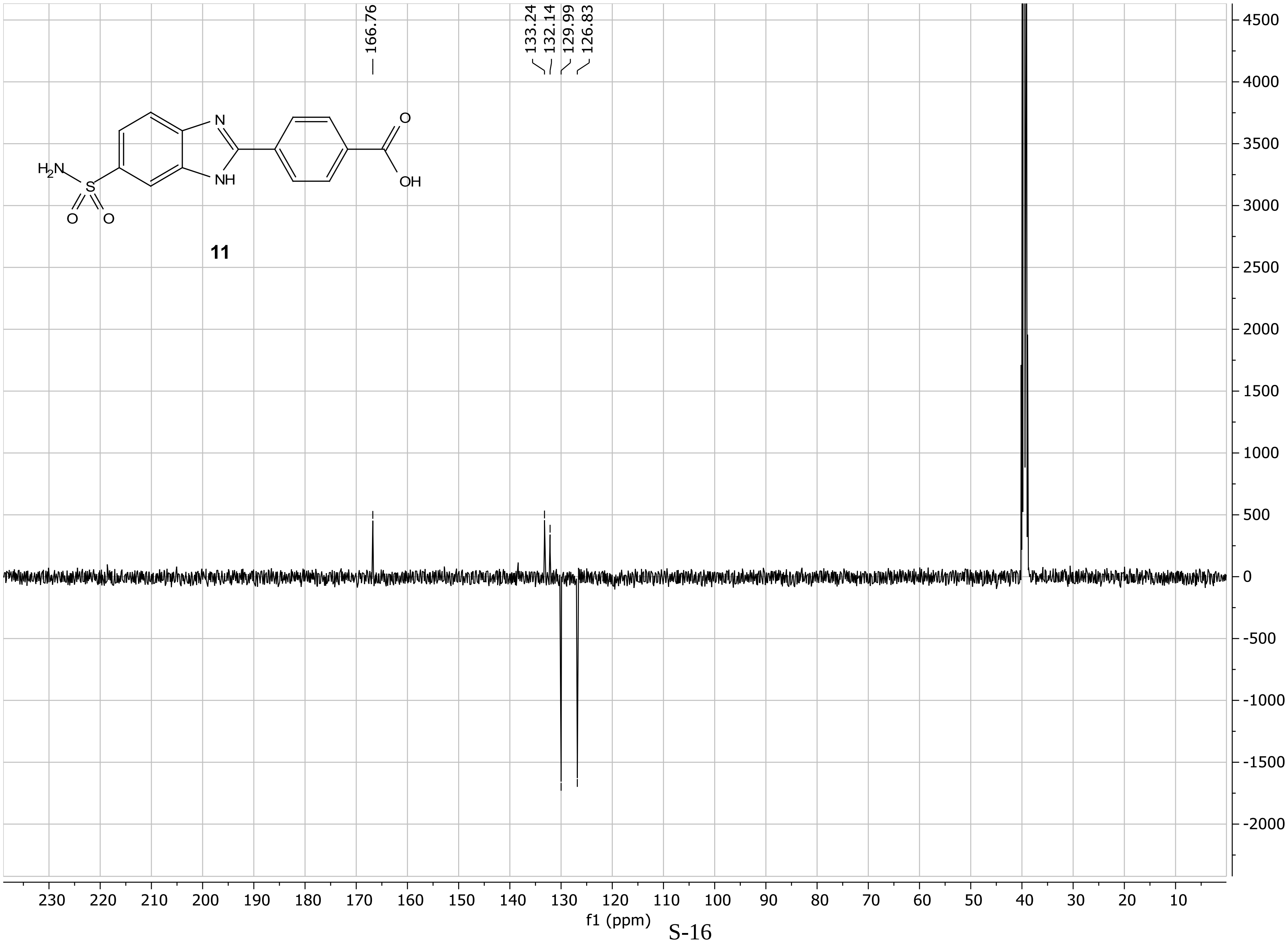

166.76
133.24
132.14
129.99
126.83
H_2N
S
O
O
N
NH
O
OH
11
4500
4000
3500
3000
2500
2000
1500
1000
500
0
-500
-1000
-1500
-2000
230 220 210 200 190 180 170 160 150 140 130 120 110 100 90 80 70 60 50 40 30 20 10
f1 (ppm)

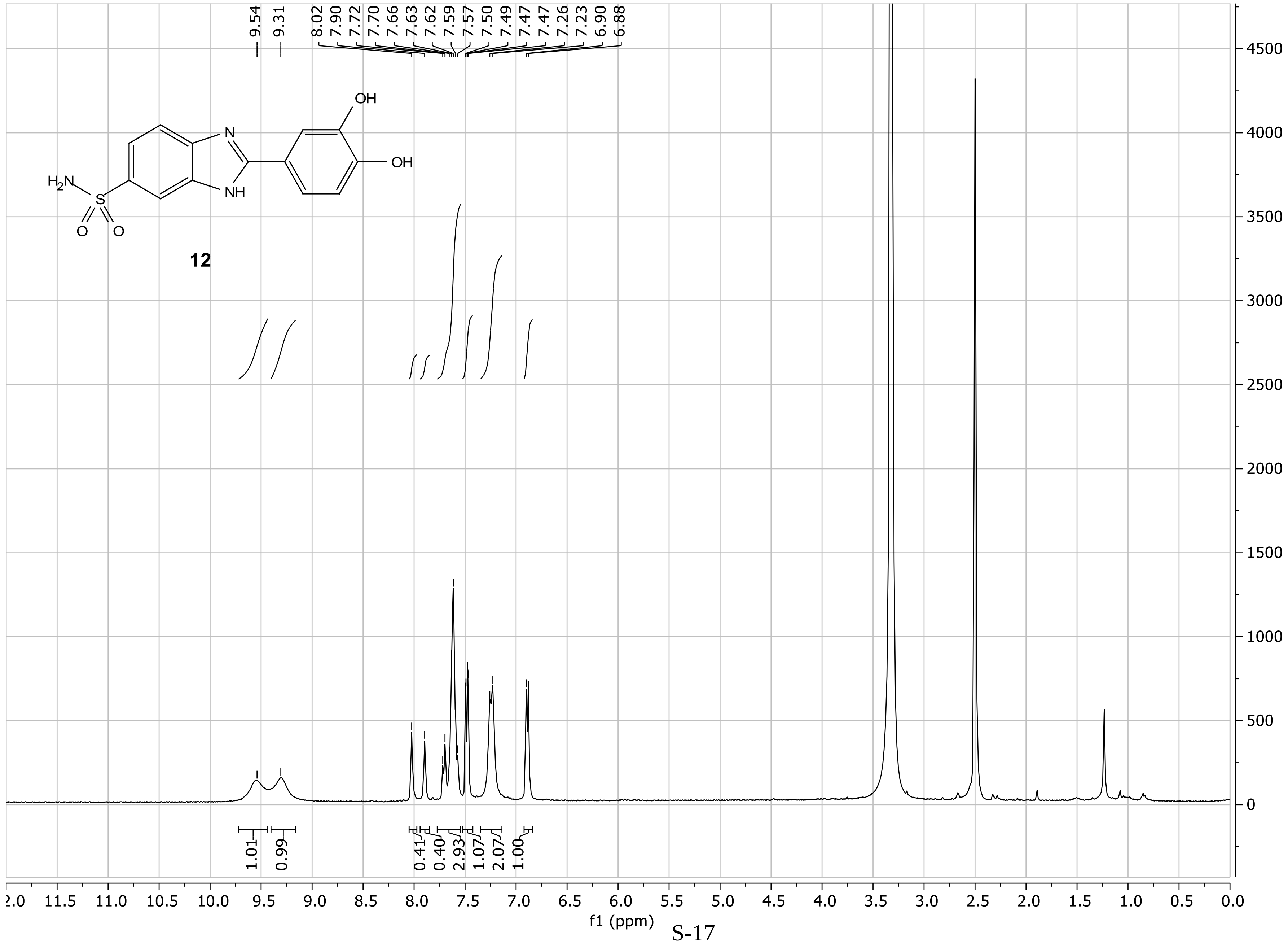
9.54
9.31
8.02
7.90
7.72
7.70
7.66
7.63
7.62
7.59
7.57
7.50
7.49
7.47
7.47
7.26
7.23
6.90
6.88
OH
OH
N
NH
H2N
S
O
O
12
1.01
0.99
0.41
0.40
2.93
1.07
2.07
1.00
f1 (ppm)

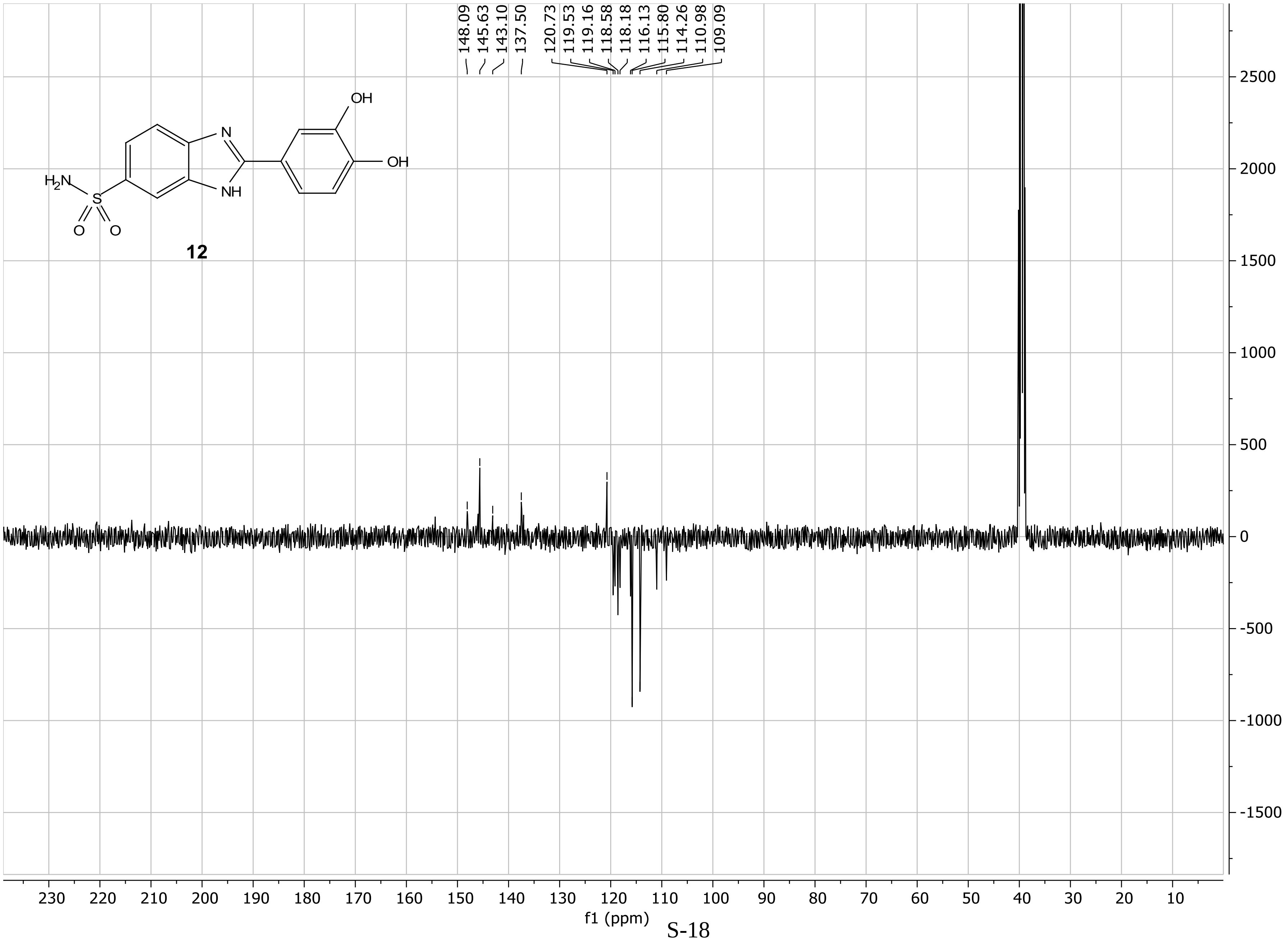

148.09
145.63
143.10
137.50
120.73
119.53
119.16
118.58
118.18
116.13
115.80
114.26
110.98
109.09
OH
OH
N
NH
H_2N
S
O
O
12
2500
2000
1500
1000
500
0
-500
-1000
-1500
230 220 210 200 190 180 170 160 150 140 130 120 110 100 90 80 70 60 50 40 30 20 10
f1 (ppm)

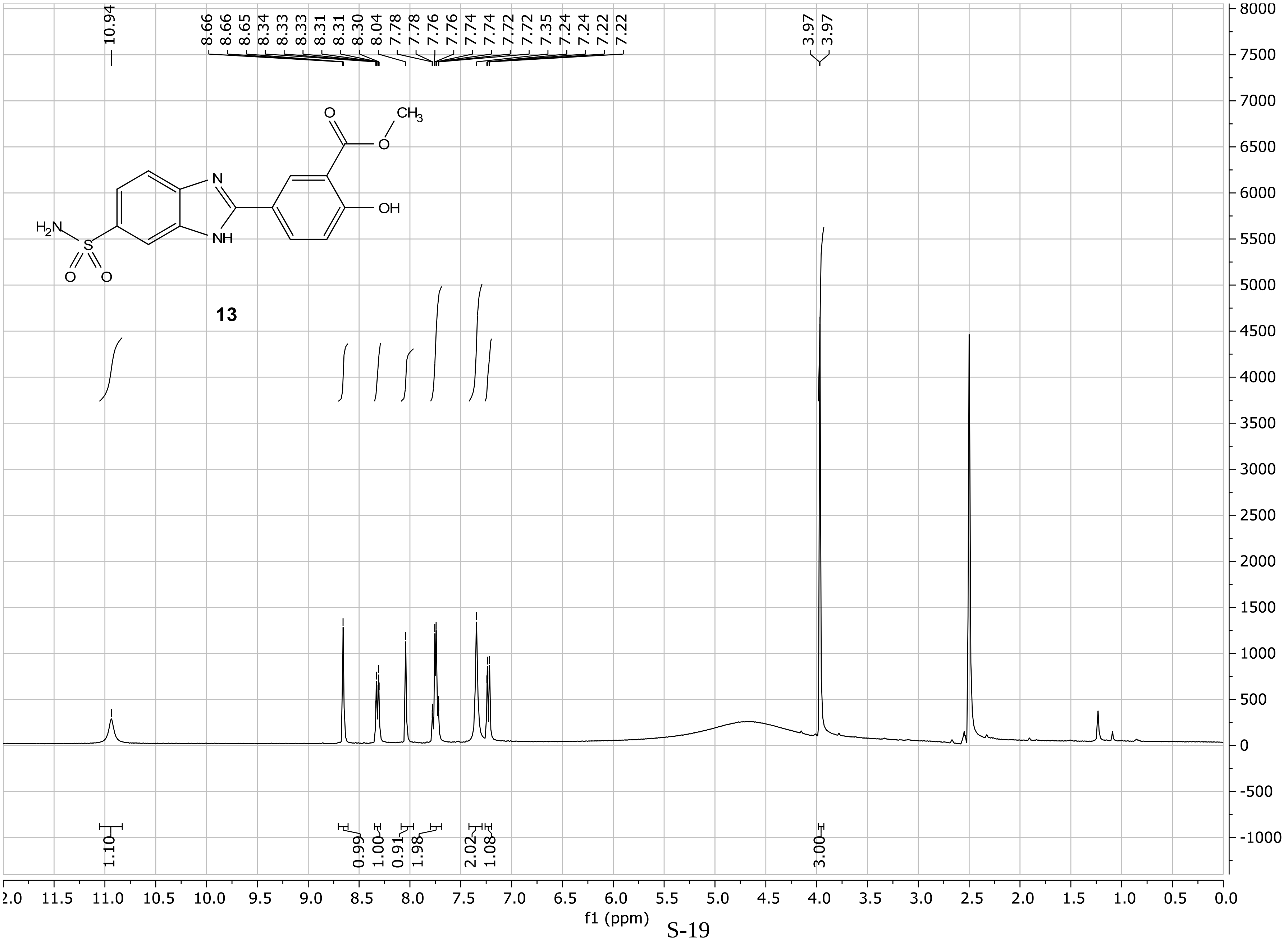
10.94
8.66
8.66
8.65
8.34
8.33
8.33
8.31
8.31
8.30
8.04
7.78
7.78
7.76
7.76
7.74
7.74
7.72
7.72
7.35
7.24
7.24
7.22
7.22
3.97
3.97
13
1.10
0.99
1.00
0.91
1.98
2.02
1.08
3.00
f1 (ppm)

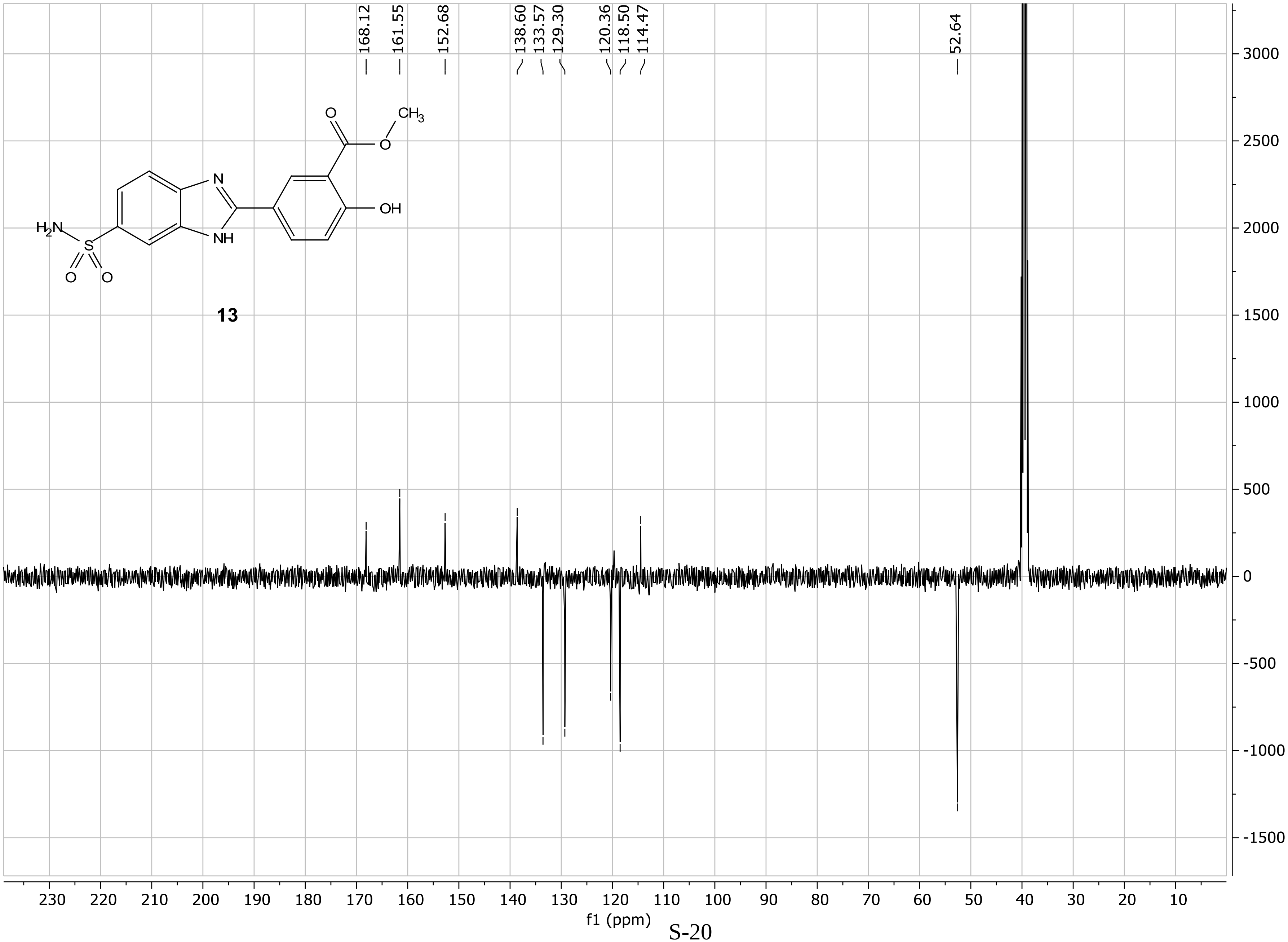
168.12
161.55
152.68
138.60
133.57
129.30
120.36
118.50
114.47
52.64
CH3
O
O
N
NH
OH
H2N
S
O
O
13
3000
2500
2000
1500
1000
500
0
-500
-1000
-1500
230 220 210 200 190 180 170 160 150 140 130 120 110 100 90 80 70 60 50 40 30 20 10
f1 (ppm)

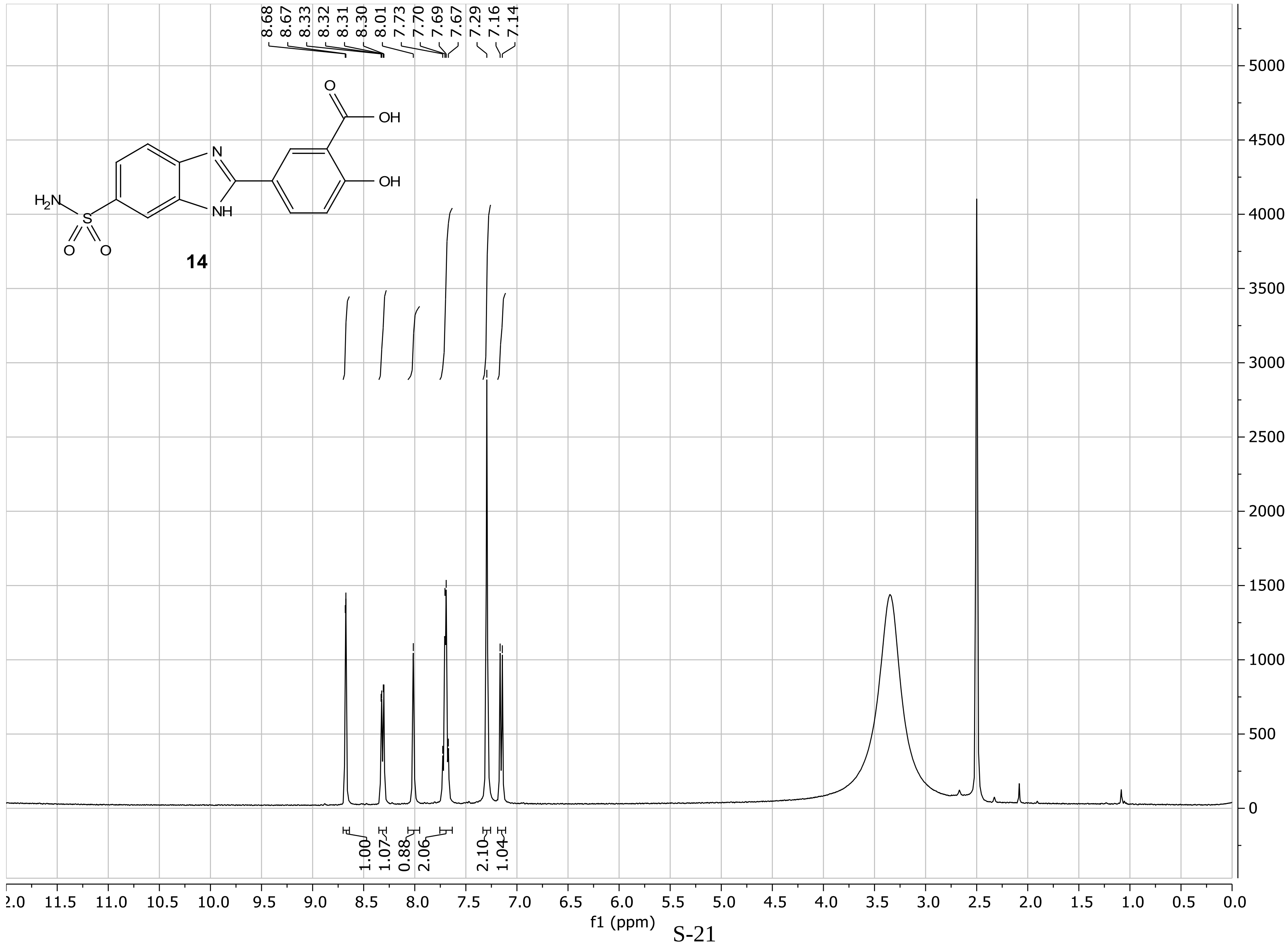
8.68
8.67
8.33
8.32
8.31
8.30
8.01
7.73
7.70
7.69
7.67
7.29
7.16
7.14
O
OH
N
OH
H_2N
NH
S
O
O
14
1.00
1.07
0.88
2.06
2.10
1.04
f1 (ppm)

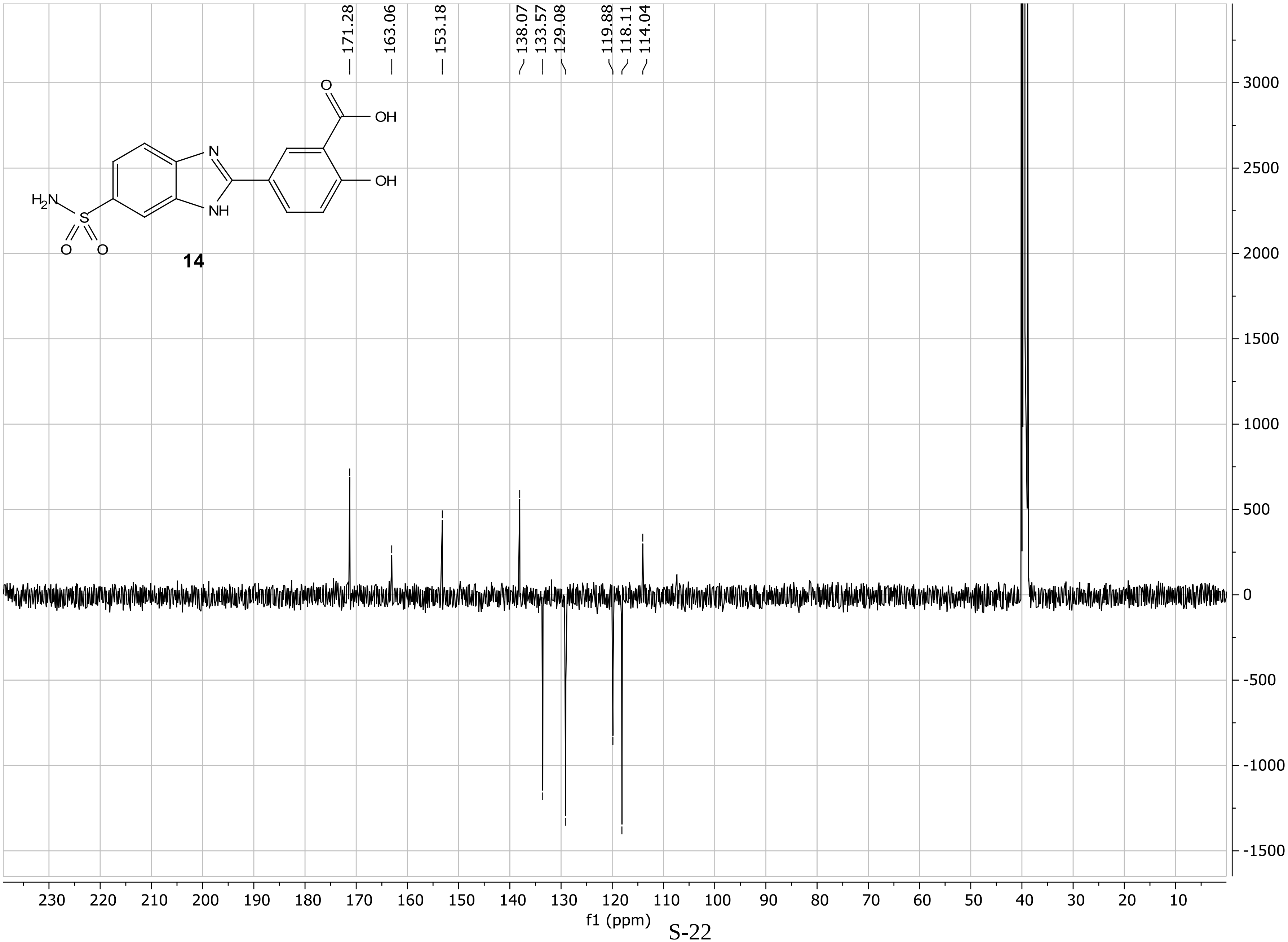
171.28
163.06
153.18
138.07
133.57
129.08
119.88
118.11
114.04
OH
OH
H2N
N
NH
S
O
O
O
14
3000
2500
2000
1500
1000
500
0
-500
-1000
-1500
230 220 210 200 190 180 170 160 150 140 130 120 110 100 90 80 70 60 50 40 30 20 10
f1 (ppm)

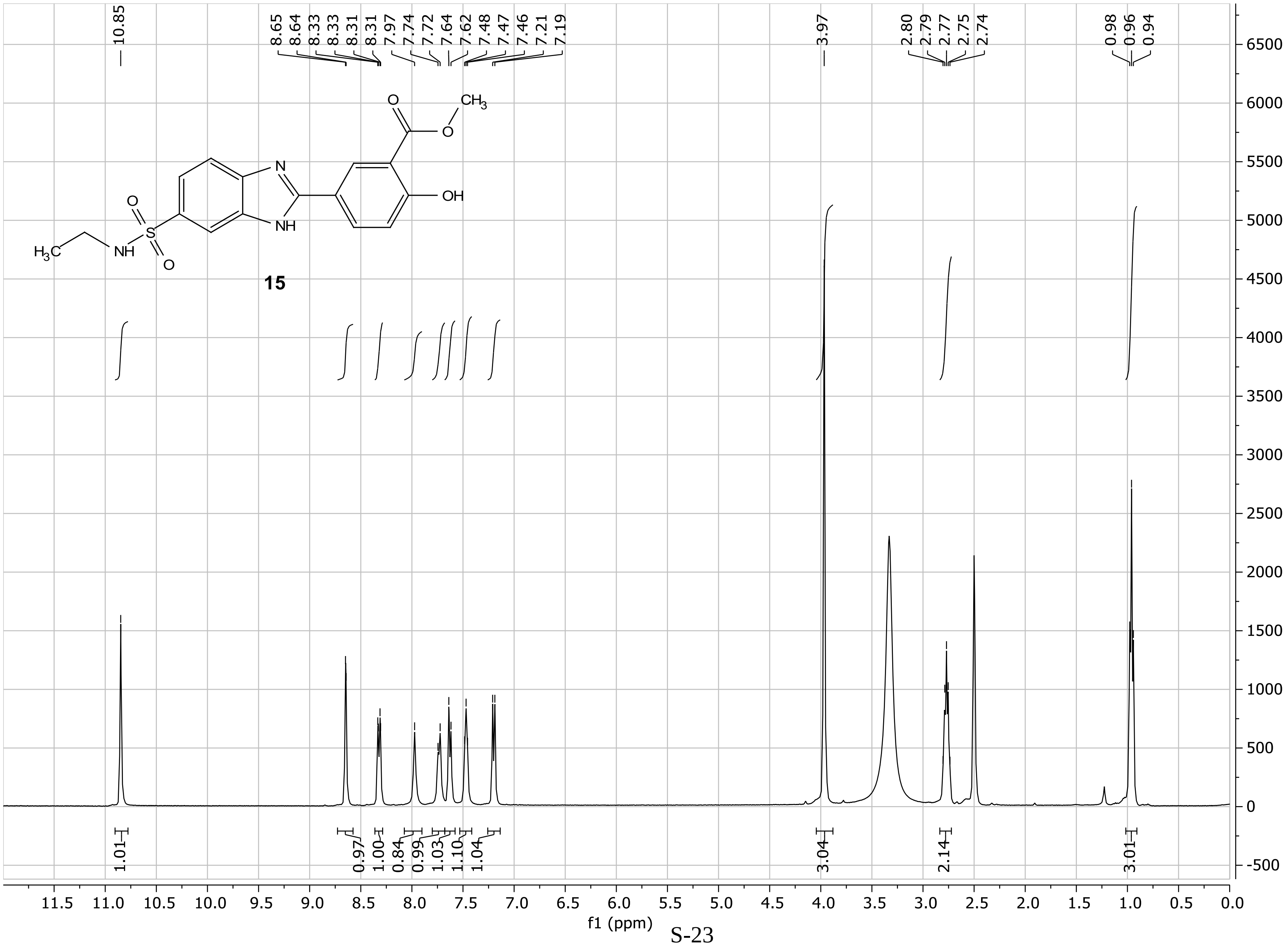

10.85
8.65 8.64 8.33 8.33 8.31 8.31 7.97 7.74 7.72 7.64 7.62 7.48 7.47 7.46 7.21 7.19
3.97
2.80 2.79 2.77 2.75 2.74
0.98 0.96 0.94
O
CH3
O
N
OH
O
S
NH
H3C
NH
O
15
1.01
0.97
1.00
0.84
0.99
1.03
1.10
1.04
3.04
2.14
3.01
f1 (ppm)

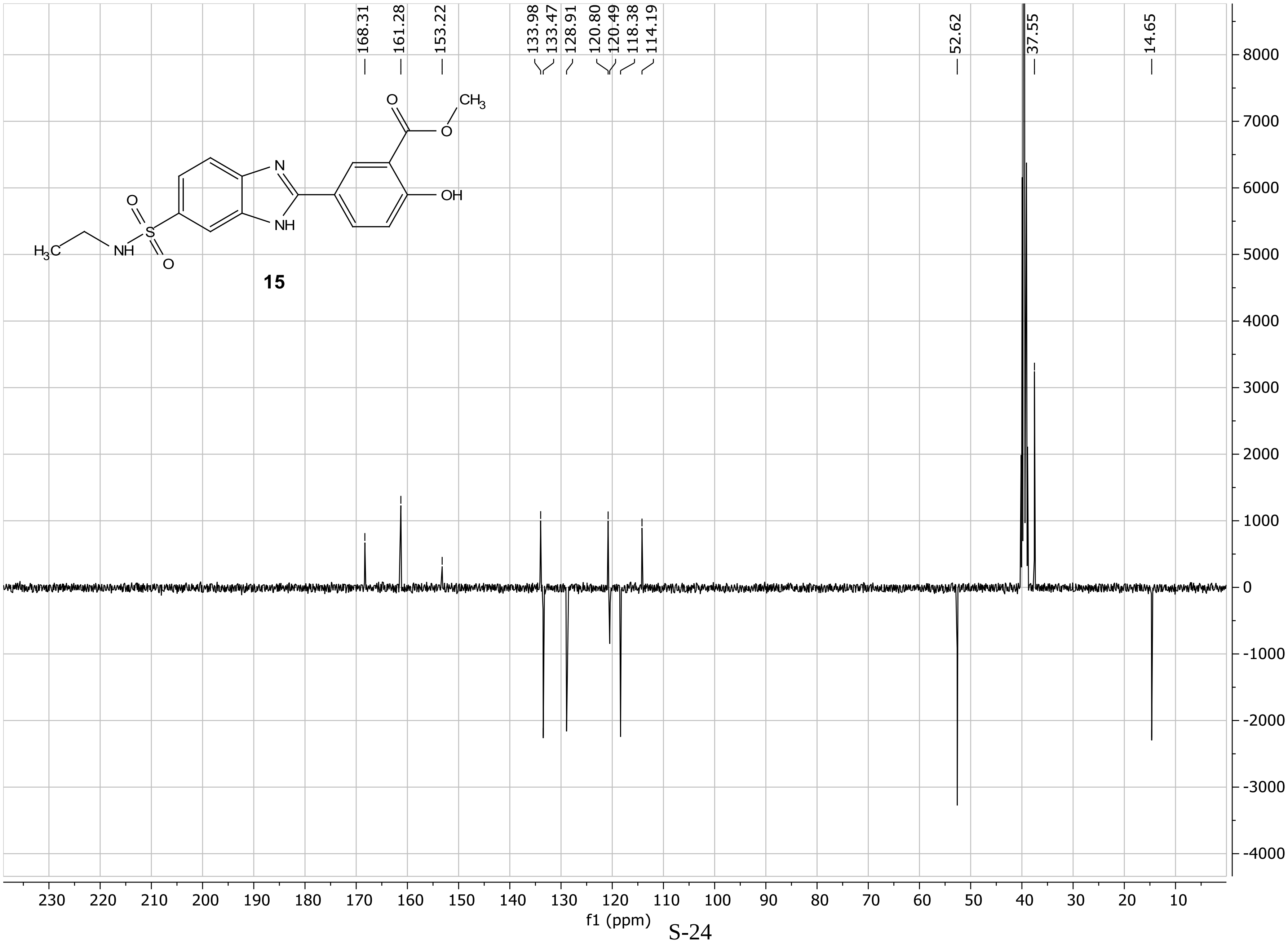
168.31
161.28
153.22
133.98
133.47
128.91
120.80
120.49
118.38
114.19
52.62
37.55
14.65
O
CH3
O
N
OH
O
S
NH
H3C
NH
O
15
8000
7000
6000
5000
4000
3000
2000
1000
0
-1000
-2000
-3000
-4000
230
220
210
200
190
180
170
160
150
140
130
120
110
100
90
80
70
60
50
40
30
20
10
f1 (ppm)

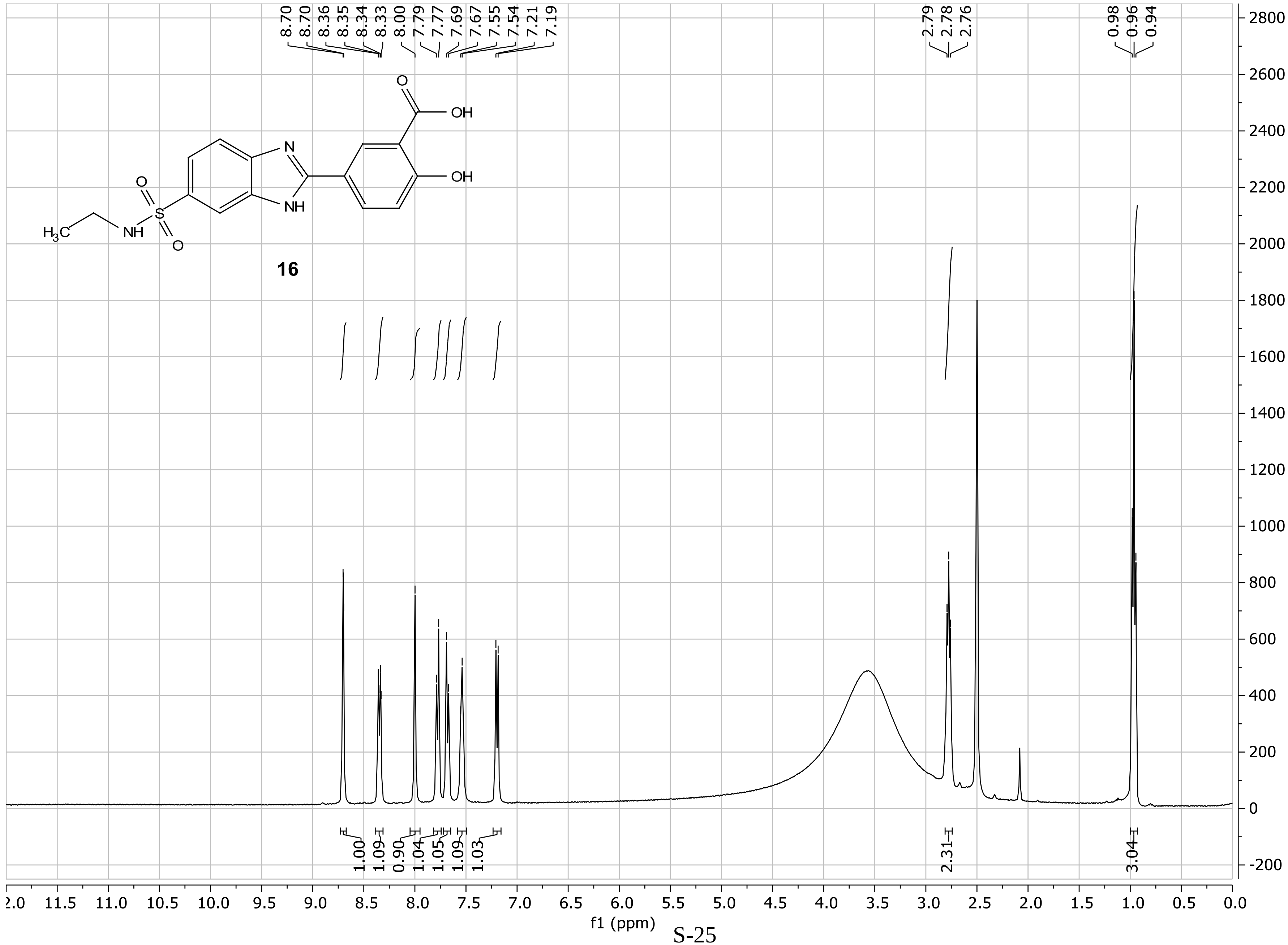

8.70 8.70 8.36 8.35 8.34 8.33 8.00 7.79 7.77 7.69 7.67 7.55 7.54 7.21 7.19
2.79 2.78 2.76
0.98 0.96 0.94
O
OH
N
OH
O
NH
H3C
NH
S
O
16
1.00 1.09 0.90 1.04 1.05 1.09 1.03
2.31
3.04
2800 2600 2400 2200 2000 1800 1600 1400 1200 1000 800 600 400 200 0 -200
11.5 11.0 10.5 10.0 9.5 9.0 8.5 8.0 7.5 7.0 6.5 6.0 5.5 5.0 4.5 4.0 3.5 3.0 2.5 2.0 1.5 1.0 0.5 0.0
f1 (ppm)

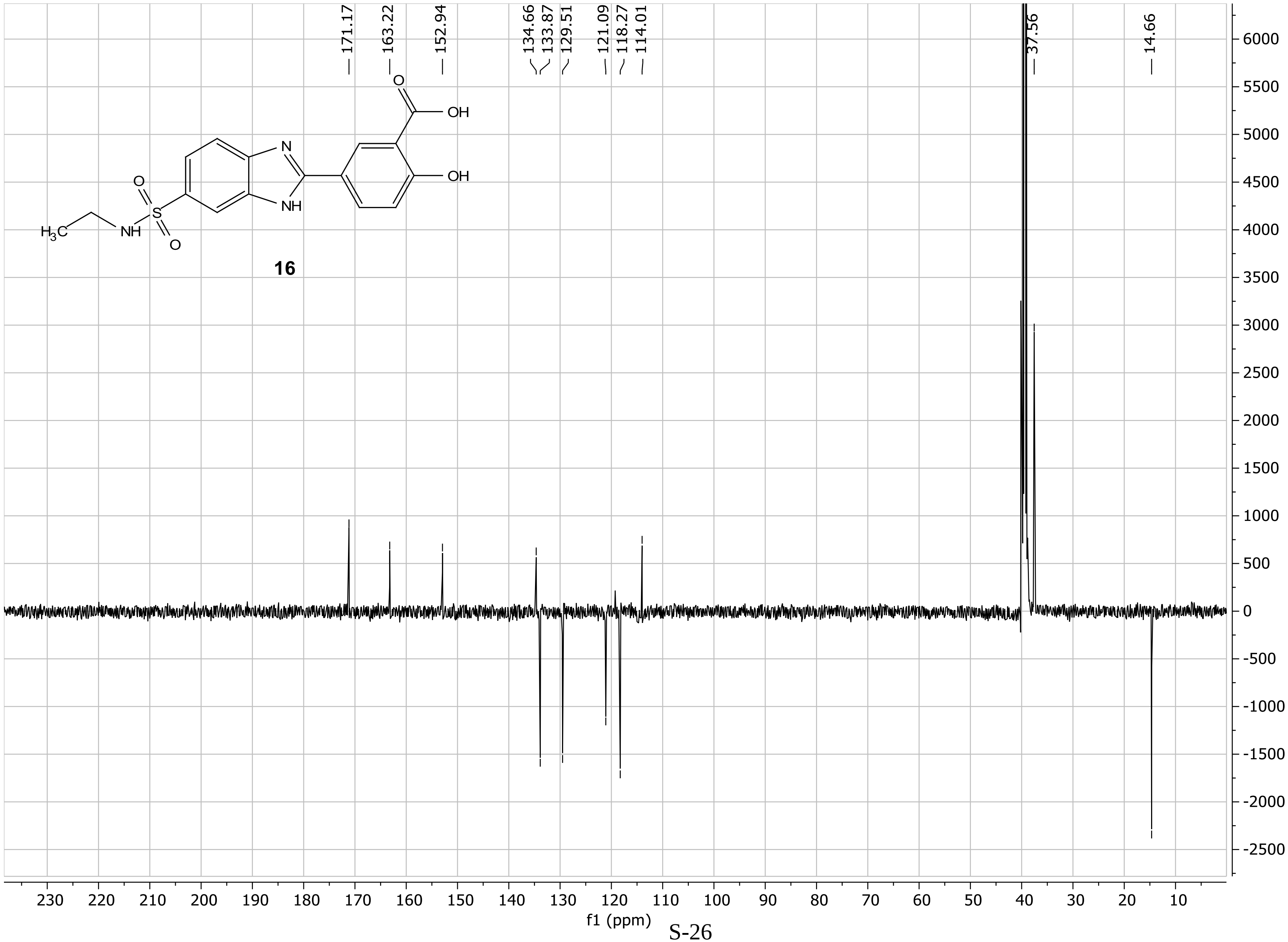

171.17
163.22
152.94
134.66
133.87
129.51
121.09
118.27
114.01
37.56
14.66
O
OH
N
OH
O
NH
S
H_3C
NH
O
16
f1 (ppm)

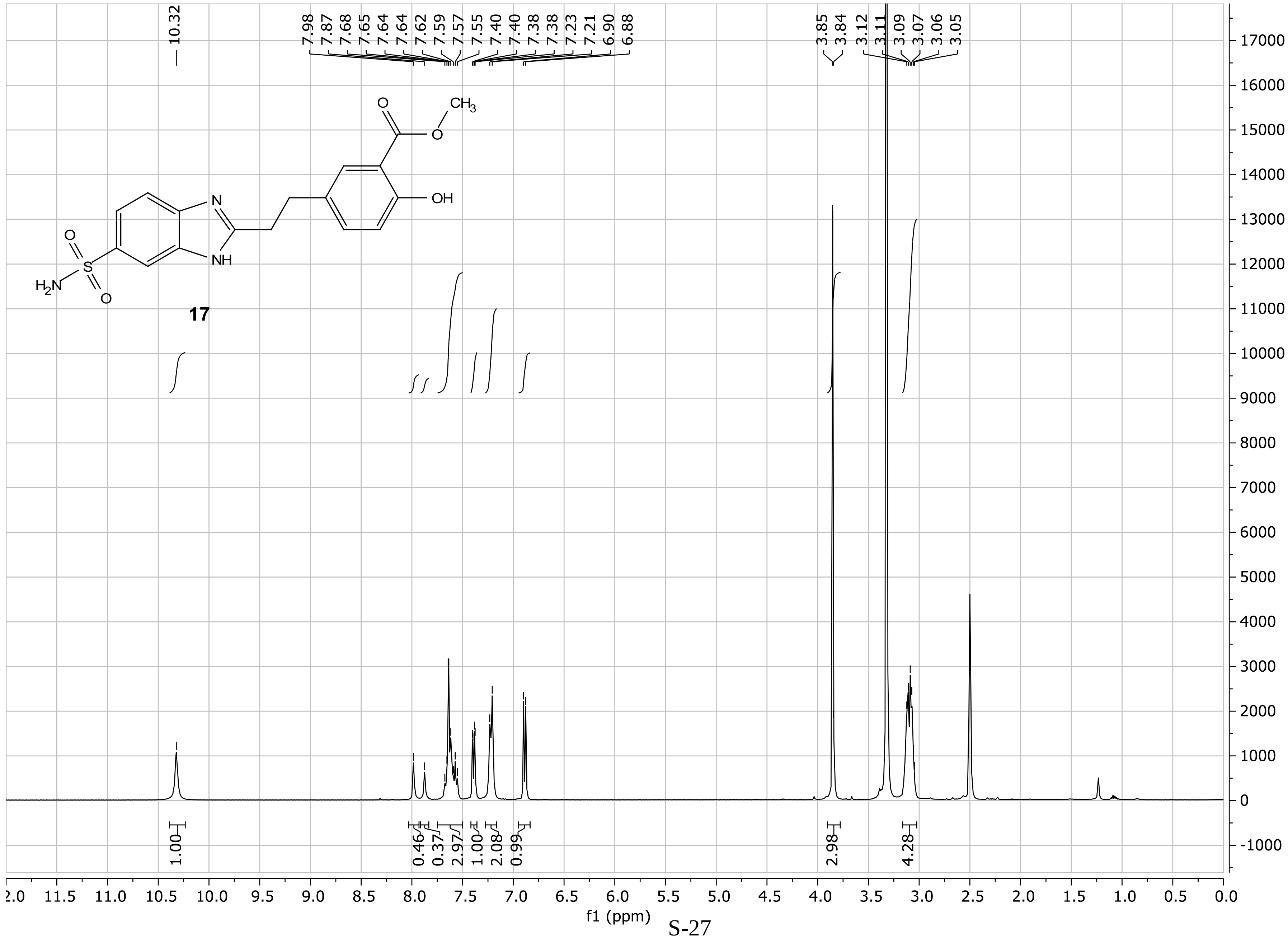
10.32
7.98
7.87
7.68
7.65
7.64
7.64
7.62
7.59
7.57
7.55
7.40
7.40
7.38
7.38
7.23
7.21
6.90
6.88
3.85
3.84
3.12
3.11
3.09
3.07
3.06
3.05
O
CH3
O
OH
N
NH
O
S
H2N
O
17
1.00
0.46
0.37
2.97
1.00
2.08
0.99
2.98
4.28
f1 (ppm)

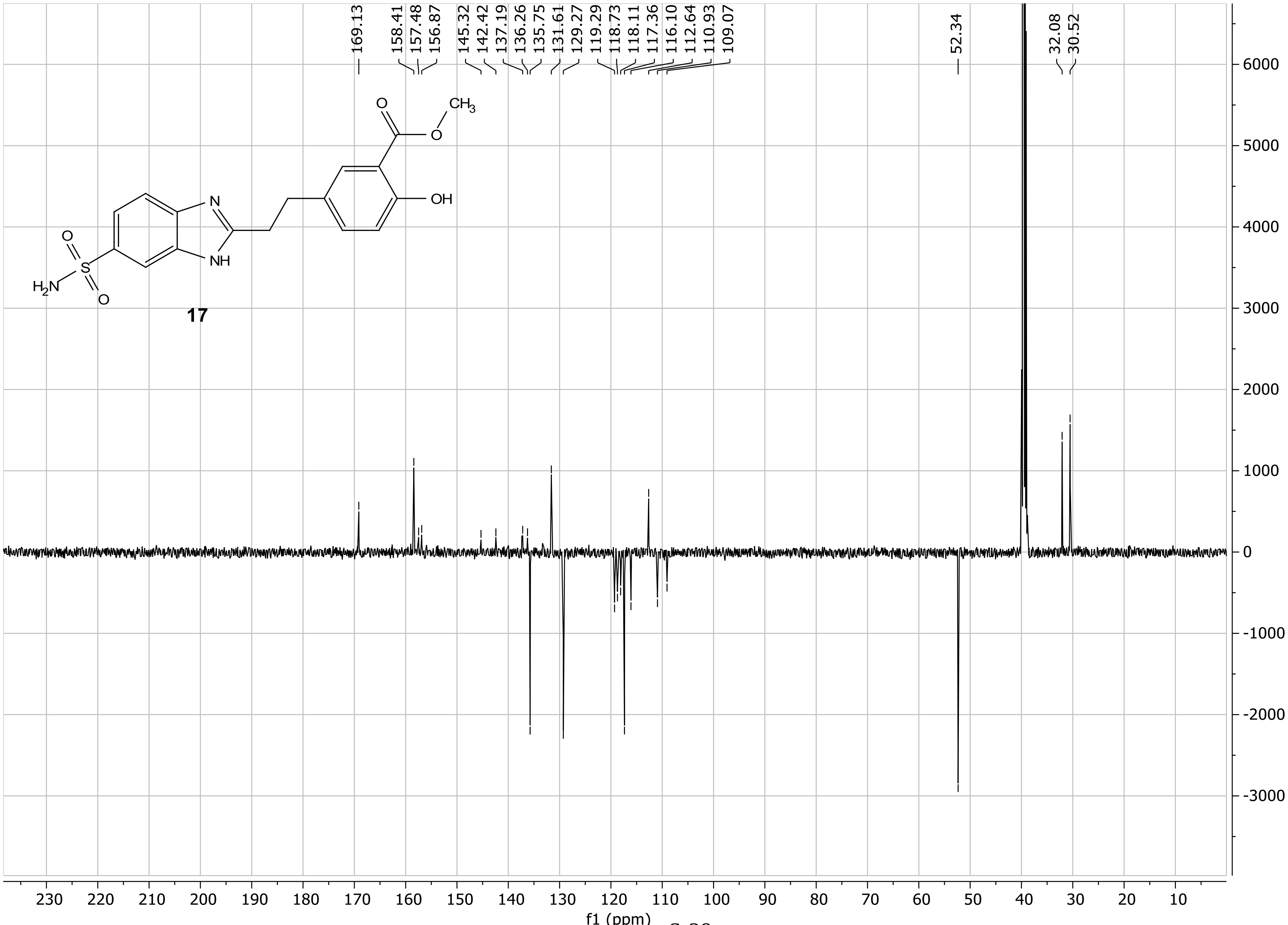

169.13
158.41
157.48
156.87
145.32
142.42
137.19
136.26
135.75
131.61
129.27
119.29
118.73
118.11
117.36
116.10
112.64
110.93
109.07
52.34
32.08
30.52
O
CH_3
O
N
OH
NH
O
S
H_2N
O
17
6000
5000
4000
3000
2000
1000
0
-1000
-2000
-3000
230
220
210
200
190
180
170
160
150
140
130
120
110
100
90
80
70
60
50
40
30
20
10
f1 (ppm)

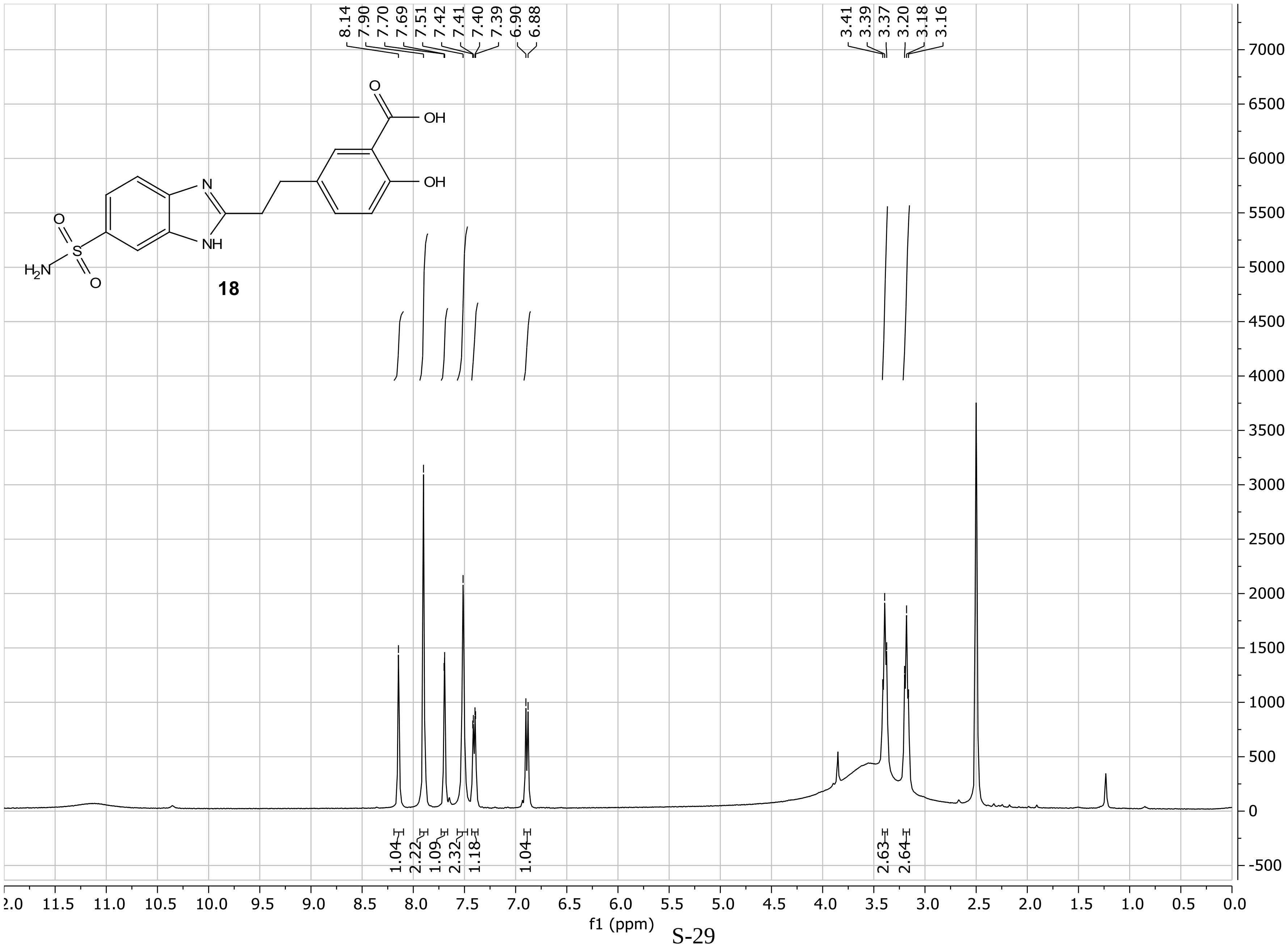
8.14
7.90
7.70
7.69
7.51
7.42
7.41
7.40
7.39
6.90
6.88
3.41
3.39
3.37
3.20
3.18
3.16
O
OH
N
OH
O
S
H2N
O
NH
18
1.04
2.22
1.09
2.32
1.18
1.04
2.63
2.64
7000
6500
6000
5500
5000
4500
4000
3500
3000
2500
2000
1500
1000
500
0
-500
2.0
11.5
11.0
10.5
10.0
9.5
9.0
8.5
8.0
7.5
7.0
6.5
6.0
5.5
5.0
4.5
4.0
3.5
3.0
2.5
2.0
1.5
1.0
0.5
0.0
f1 (ppm)

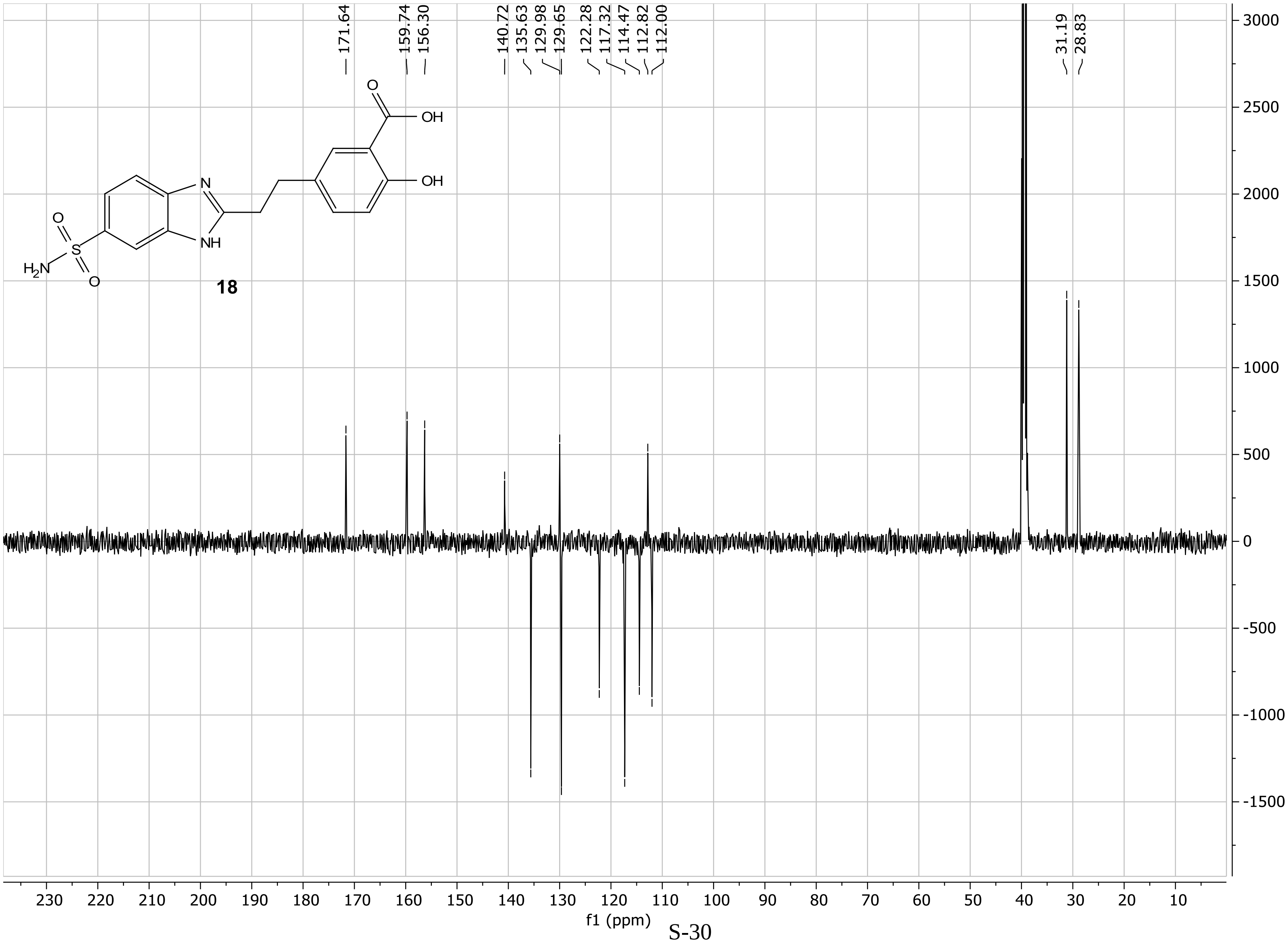
171.64
159.74
156.30
140.72
135.63
129.98
129.65
122.28
117.32
114.47
112.82
112.00
31.19
28.83
O
OH
N
OH
O
S
H2N
O
NH
18
3000
2500
2000
1500
1000
500
0
-500
-1000
-1500
230
220
210
200
190
180
170
160
150
140
130
120
110
100
90
80
70
60
50
40
30
20
10
f1 (ppm)

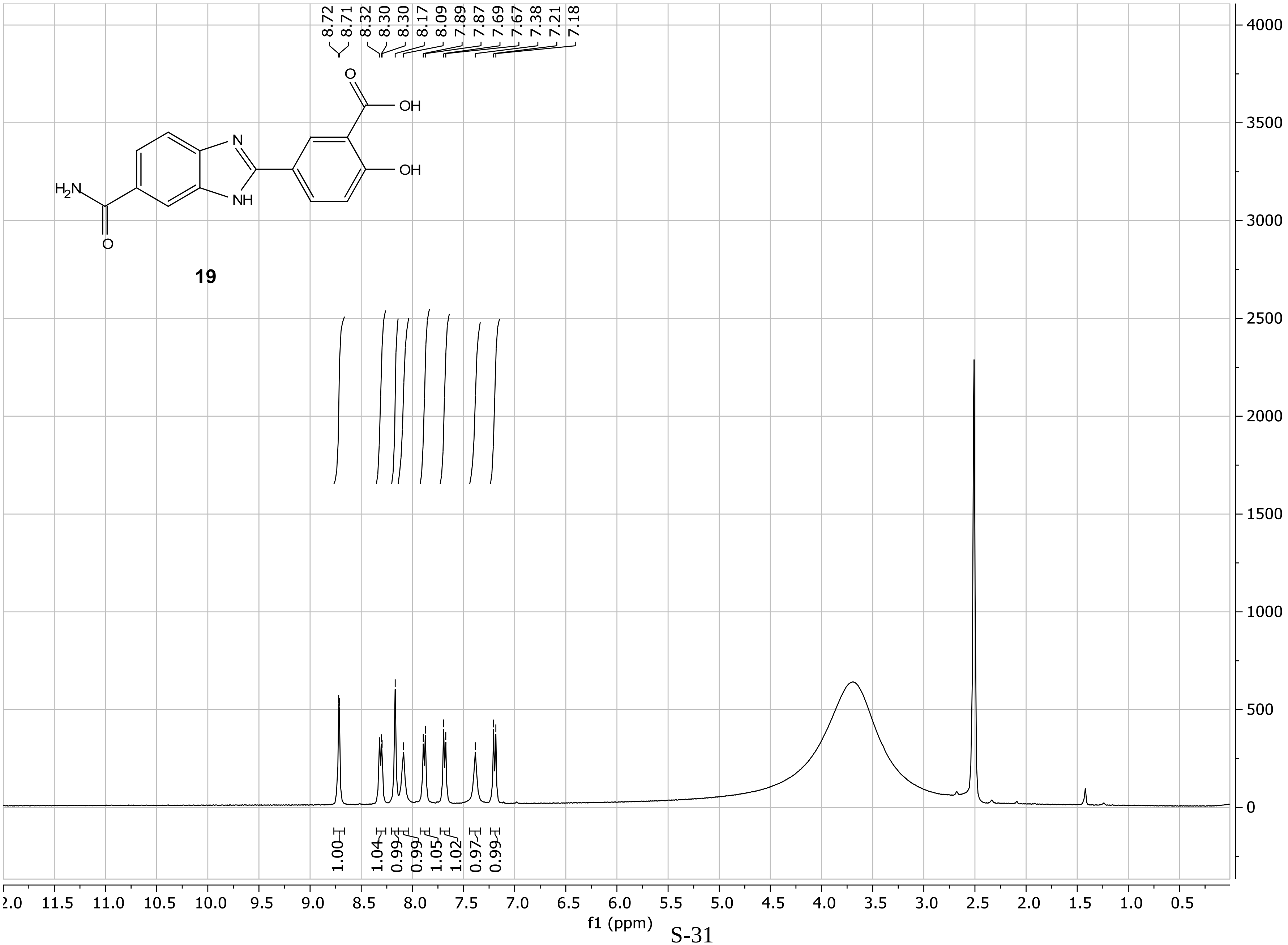
8.72
8.71
8.32
8.30
8.30
8.17
8.09
7.89
7.87
7.69
7.67
7.38
7.21
7.18
19
1.00
1.04
0.99
0.99
1.05
1.02
0.97
0.99
f1 (ppm)

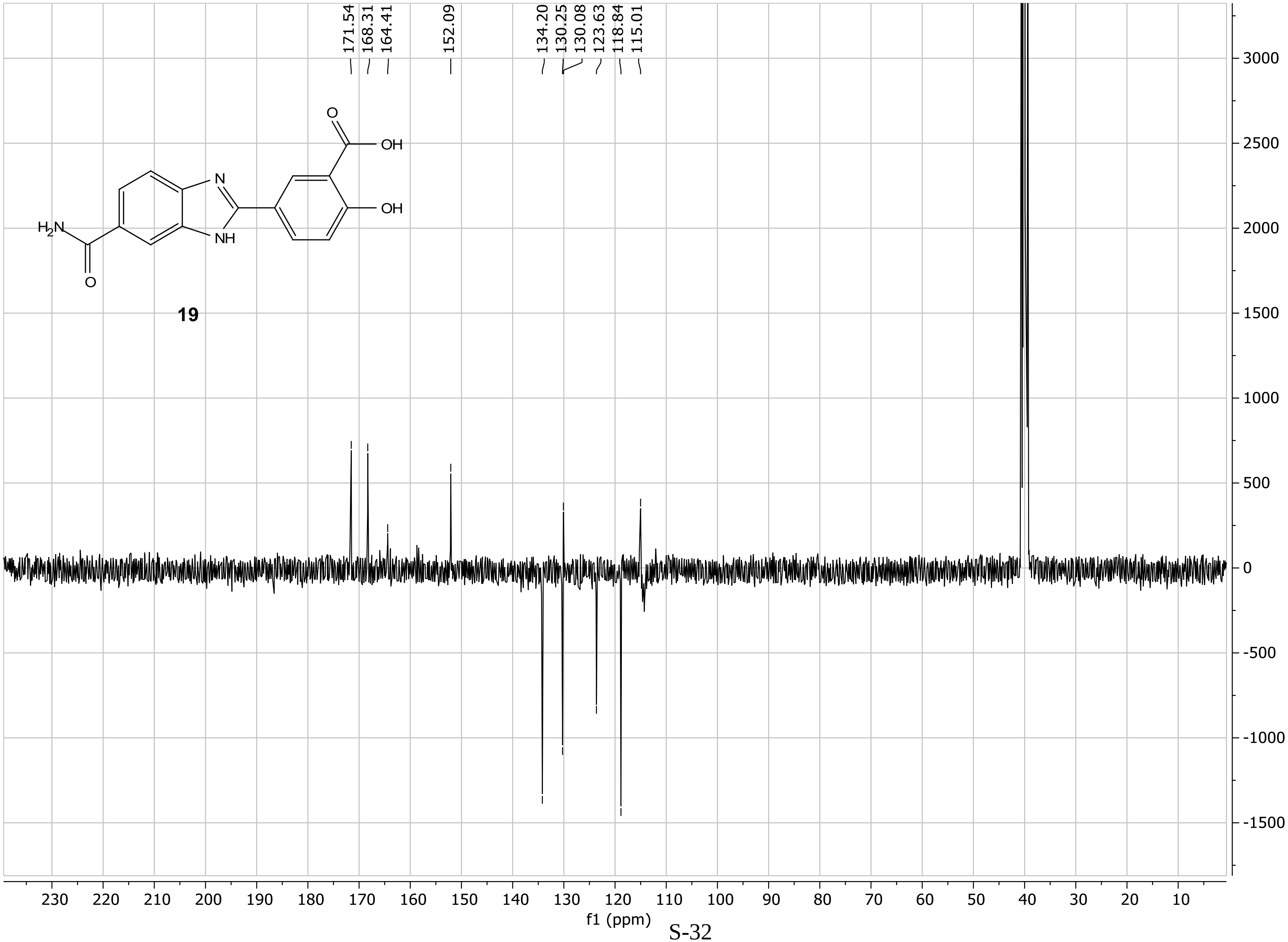
171.54
168.31
164.41
152.09
134.20
130.25
130.08
123.63
118.84
115.01
19
f1 (ppm)
